# Recycling oyster shell waste as a bio-derived pore-promoting additive for porous phosphate-based geopolymers produced from volcanic slag

**DOI:** 10.1039/d6ra08514h

**Published:** 2026-09-28

**Authors:** Steve Keyrane Kuida Tchikapa, Priscilla S. Brunetto, Antoine Scalabre, Placide Desiré Belibi Belibi, Katharina M. Fromm, Julius Ndi Nsami, Antoine Elimbi

**Affiliations:** a Applied Physical and Analytical Chemistry Laboratory, Department of Inorganic Chemistry, University of Yaoundé I Yaoundé Cameroon beplacide85@gmail.com keyranekuida70@gmail.com; b Department of Chemistry, University of Fribourg Chemin du Musée, 9 1700 Fribourg Switzerland; c Applied Inorganic Chemistry Laboratory, Department of Inorganic Chemistry, University of Yaoundé I Yaoundé Cameroon

## Abstract

Porous phosphate-based geopolymers were synthesized from volcanic slag using calcined oyster shell waste (COS) as a bio-derived calcium source and pore-development promoting additive. Volcanic slag was partially substituted with 0, 5, 10, and 15 wt% COS and activated with phosphoric acid solutions of 6 and 10 mol L^−1^. The influence of COS incorporation on phase evolution, microstructure development, and engineering performance was investigated using XRD, FTIR, SEM/EDS, TGA/SDTA, and physicomechanical analyses. The results revealed that phosphoric acid concentration and calcium availability jointly controlled precursor dissolution and reaction-product formation. Calcium released from COS promoted the precipitation of calcium phosphate phases, predominantly monetite (CaHPO_4_), while phosphoric acid activation favored the formation of a continuous aluminosilicophosphate matrix. The balance between these competing processes governed pore development and matrix continuity. Among the investigated formulations, the 5 wt% COS composition generated the most favorable balance between homogeneous pore development and compressive strength (21.74 MPa compared with 22.85 MPa for the reference formulation). Higher COS contents increased microstructural heterogeneity, efflorescence, and strength loss. This study provides mechanistic insights into pore formation in phosphate-based geopolymers and demonstrates a promising waste-valorization route for converting oyster shell waste and volcanic slag into functional porous materials.

## Abstract 2

Calcined oyster shells promote pore development associated with calcium phosphate formation. At 5 wt% COS, a homogeneous porous structure is achieved while largely preserving compressive strength.

## Graphical abstract

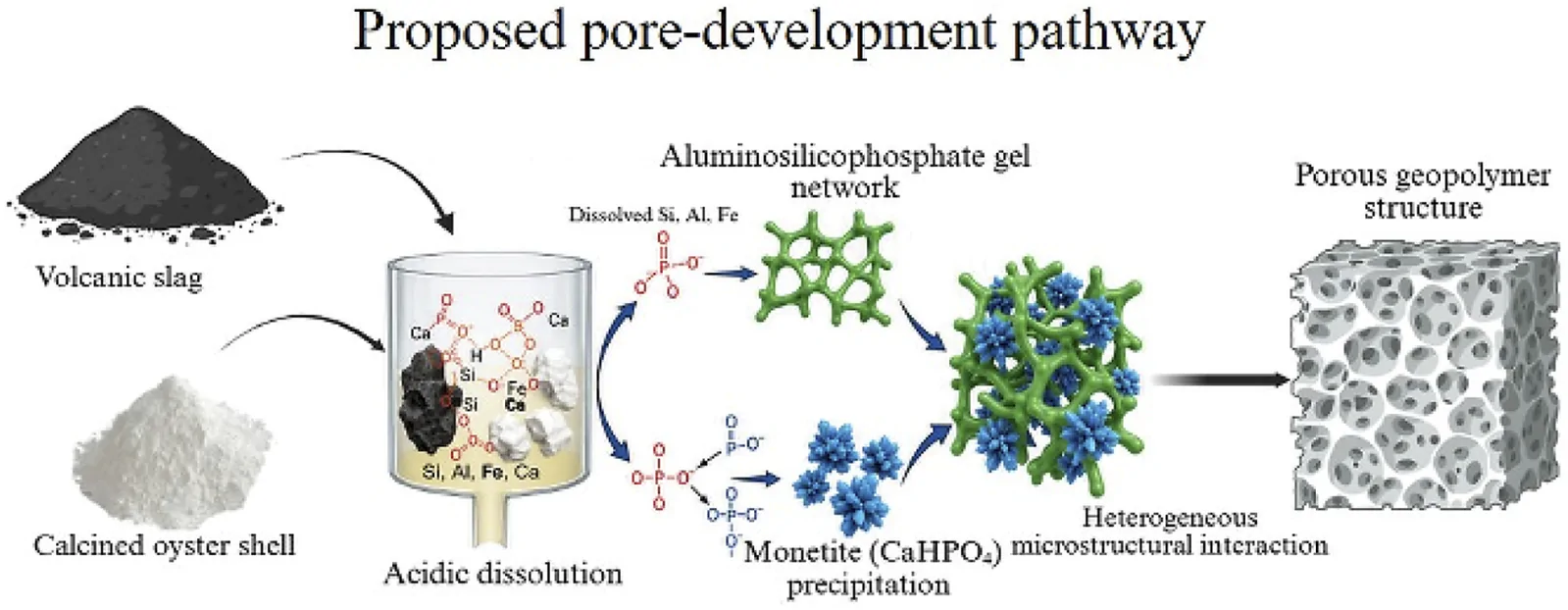

## 1 Introduction

Geopolymers are inorganic aluminosilicate binders produced through the chemical activation of reactive aluminosilicate sources and generally described by the empirical formula M_2_O·Al_2_O_3_·4SiO_4_·*n*H_2_O, where M denotes an alkali metal and *n* represents the amount of water incorporated within the geopolymeric structure.^1^ These materials can be synthesized from a wide variety of natural minerals and industrial by-products, including metakaolin derived from clay calcination,^2–5^ metallurgical slag, rice husk ash and red mud,^6,7^ fly ash^8–11^ and volcanic slag (VS).^12–14^ Their nanostructure exhibits similarities to that of zeolitic frameworks but remains predominantly amorphous owing to the absence of long-range crystallographic order.^2,15,16^

Depending on the nature of the activating medium, geopolymers are generally classified into alkali-activated and acid-activated systems. Alkali-activated geopolymers are mainly composed of Si–O–Al linkages, whereas phosphate-based geopolymers synthesized in acidic media contain structural units such as Al–O–P–O, P–O–Si–O–P–O, P–O–P–O and P–O–Si–O–Al–O–P–O.^17^ The reaction products of phosphate geopolymers have been reported to consist primarily of amorphous SiO_2_·Al_2_O_3_·P_2_O_5_·*n*H_2_O gels and hydrated aluminophosphate species.^18^

Compared with alkali-activated counterparts, phosphate geopolymers frequently exhibit superior compressive strength, thermal resistance and dielectric properties while offering a lower-carbon alternative to conventional Portland cement-based materials.^3,11,17,19–21^ In recent years, porous geopolymers have attracted considerable scientific and technological interest because they combine chemical stability, mechanical robustness, high specific surface area and interconnected pore networks. These characteristics make them attractive for a broad range of environmental and engineering applications. Porous geopolymeric materials can be produced by several methods, including direct foaming, replica processes, sacrificial templating and additive manufacturing approaches. Among these routes, direct foaming remains particularly attractive because of its simplicity and industrial scalability. The process relies on the incorporation of blowing agents capable of generating gas bubbles within the geopolymeric matrix during setting and hardening. A wide variety of pore-forming agents have been investigated, including CaCO_3_ and CaO,^5,20,21,23^ CH_3_COOH,^6^ CaSO_4_,^8^ H_2_O_2_,^9,22,24^ SiO_2_,^11,19^ and metallic Al or Fe powders.^25,26^ The resulting porous materials have been investigated for applications such as dye removal,^22,24,27,28^ heavy-metal adsorption,^29–32^ antibacterial water treatment^2,33,34^ and photocatalytic remediation processes.^35–37^ However, the suitability of a given porous geopolymer for these applications depends on its specific pore structure, physicochemical properties, and functional performance. Moreover, many conventional blowing agents remain relatively expensive, highly reactive, or difficult to control during pore generation, which may result in poor pore homogeneity and substantial losses in mechanical performance. Consequently, the search for abundant, low-cost and environmentally sustainable pore-forming agents remains a key challenge for the large-scale development of porous geopolymer technologies.

Volcanic slag (VS) constitutes a particularly attractive precursor for geopolymer synthesis because of its high content of SiO_2_, Al_2_O_3_ and Fe_2_O_3_, whose combined concentration generally exceeds 70 wt%, satisfying the compositional requirements specified by ASTM C618 for reactive pozzolanic materials.^38^ Despite its excellent geopolymerization potential, VS remains significantly less investigated than metakaolin or fly ash due to its uneven geographical distribution. Cameroon possesses extensive volcanic formations, including Mount Cameroon and Mount Manengouba, which provide abundant and compositionally diverse volcanic resources.^39,40^ Nevertheless, these materials are currently underutilized and are mainly employed as abrasive materials, ceramic additives and soil-conditioning agents.^41^

In parallel, the management of oyster shell (OS) waste represents an emerging environmental concern. Oyster shells consist of approximately 96% CaCO_3_ and have been widely investigated as low-cost calcium-rich resources for the manufacture of lime, cement, concrete, coatings, pigments, paper additives, polymer composites and biomedical materials.^42,43^ In Cameroon, oyster shells represent nearly 70% of the total oyster mass and are commonly discarded without valorization. Although they cannot be considered hazardous waste, their accumulation causes landscape degradation and inefficient land occupation. Furthermore, the decomposition of residual organic matter may generate NH_3_ and H_2_S emissions, creating environmental and sanitary concerns for nearby populations.^44,45^

Calcination transforms oyster shells into a highly reactive CaO-rich material capable of rapidly reacting with phosphoric acid. Previous studies have demonstrated that calcium-rich materials can simultaneously contribute to pore generation and modify the geopolymerization mechanisms of phosphate-activated systems through the precipitation of calcium phosphate phases.^20,21^ However, despite the abundance, low cost and high calcium content of oyster shell waste, its use in phosphoric acid-activated volcanic slag geopolymers remains largely unexplored. Furthermore, the influence of oyster shell-derived calcium on the balance between calcium phosphate precipitation, matrix development, microstructural evolution and mechanical performance has not been systematically investigated. This knowledge gap is particularly important because calcium species may exert a dual effect during geopolymerization. On one hand, they can promote pore formation through rapid precipitation reactions with phosphate species; on the other hand, excessive calcium phosphate formation may compete with the development of the three-dimensional aluminosilicophosphate network and adversely affect the integrity of the resulting material. Therefore, establishing the optimal level of calcium incorporation capable of maximizing porosity while preserving mechanical performance remains an unresolved scientific issue.

In this context, the present study investigates the feasibility of utilizing calcined oyster shells (COS) as a bio-derived calcium-rich additive capable of promoting pore development for the production of porous phosphate-based geopolymers from volcanic slag and explores their potential for waste valorization. Volcanic slag was partially substituted with 0, 5, 10 and 15 wt% COS and activated using phosphoric acid solutions at concentrations of 6 and 10 mol L^−1^. The precursors were characterized by XRF, TGA/SDTA, FT-IR, XRD and BET-BJH analyses, while the synthesized geopolymers were investigated using FT-IR, TGA/SDTA, XRD, SEM/EDS, setting time, water absorption, linear shrinkage and compressive strength measurements. Particular attention was devoted to elucidating the relationships between precursor dissolution, calcium phosphate precipitation, pore formation, microstructural evolution and the resulting engineering properties of the porous geopolymers.

## 2 Materials and methods

### 2.1 Raw materials

Volcanic slag (VS) collected from the Adamawa region (Cameroon) was used as the aluminosilicate precursor. Oyster shells (OS) were collected from the coastal area of Mouanko (Cameroon). Detailed information regarding the preparation, conditioning, and preliminary characterization of both materials is provided in the SI (SI) Text S1.

A commercial phosphoric acid solution (85 wt% H_3_PO_4_) was used as the activating agent. The stock solution was diluted with distilled water to obtain phosphoric acid solutions with concentrations of 6 and 10 mol L^−1^.

### 2.2 Preparation of porous geopolymers

Porous phosphate geopolymers, denoted GVS_*x*_^*y*^COS, were synthesized by partially substituting VS with calcined oyster shell (COS) at replacement levels of 0, 5, 10, and 15 wt%. In the nomenclature, *x* represents the phosphoric acid concentration (6 or 10 mol L^−1^), while *y* corresponds to the COS content (0, 5, 10, or 15 wt%). Prior to geopolymerization, VS and COS powders were dry-mixed for 3 min using a laboratory mixer (M & O, N50-G) to ensure compositional homogeneity. The phosphoric acid activating solution was then gradually added according to the liquid-to-solid (L/S) ratios listed in Table 1. It should be noted that the liquid-to-solid ratio (L/S) was adjusted to maintain workable pastes as COS content increased. Consequently, the effects discussed throughout this study reflect the combined influence of COS incorporation, activator concentration and the associated changes in formulation liquid demand. Fresh pastes were mixed for 5 min to obtain a homogeneous and workable consistency. The resulting pastes were cast into 40 × 40 × 40 mm cubic molds and manually vibrated for 3 min to remove entrapped air. Specimens were allowed to harden at ambient laboratory conditions for 48 h before demolding. The hardened samples were subsequently wrapped in polyethylene film to minimize moisture loss and cured at room temperature (23 ± 5 °C) for 28 days. For each formulation, a single homogeneous batch was prepared and subsequently used to produce the specimens required for different characterization and testing procedures. Cubic specimens (40 × 40 × 40 mm) were used for linear shrinkage, compressive-strength measurements and microstructural characterization, whereas dedicated cylindrical specimens (20 mm diameter × 40 mm height) were prepared for water-absorption testing. All physicomechanical measurements were performed on three independent specimens per formulation, and the reported values correspond to mean values. Error bars shown in the figures represent the corresponding standard deviations. The procedures employed for setting time determination, water absorption, linear shrinkage, and compressive strength measurements are described in the SI, Text S3.

**Table 1:** Mix designs of the experimented pastes developed

Activating solution concentration (mol L^−1^)	Formulations	L/S ratio	VS (% mass)	COS (%mass)
10	GVS_10_^0^COS	0.58	100	0
	GVS_10_^5^COS	0.75	95	5
	GVS_10_^10^COS	1.00	90	10
	GVS_10_^15^COS	1.20	85	15
6	GVS_6_^0^COS	0.43	100	0
	GVS_6_^5^COS	0.55	95	5
	GVS_6_^10^COS	0.73	90	10
	GVS_6_^15^COS	0.87	85	15

### 2.3 Characterization techniques

The chemical compositions of VS and OS were determined by wavelength-dispersive X-ray fluorescence spectroscopy (XRF, Malvern PANalytical Zetium). The specific surface area, pore volume, and pore-size distribution of VS were evaluated using N_2_ adsorption–desorption measurements (Micromeritics 3Flex) and calculated according to the BET and BJH methods. Thermal behavior was investigated by simultaneous thermogravimetric and differential thermal analysis (TGA/SDTA, Mettler Toledo TGA/DSC 3+) between 25 and 1000 °C at a heating rate of 10 °C min^−1^ under air atmosphere. These analyses were performed to evaluate the thermal stability of VS and determine the calcination conditions required for the conversion of CaCO_3_ contained in oyster shells into reactive CaO. Thermal analyses of the synthesized geopolymers are provided in SI (Fig. S8 and S9). Fourier-transform infrared spectroscopy (FTIR, PerkinElmer Frontier) was performed in transmittance mode over the spectral range 4000–400 cm^−1^ to identify structural changes occurring during calcination and geopolymerization. Phase assemblages were determined by X-ray diffraction (XRD, Bruker D8 Advance, CuKα radiation, *λ* = 1.5406 Å). Diffraction patterns were analyzed using Match!® software (Crystal Impact, Version 4) and compared with the Crystallography Open Database (COD) for phase identification. Particular attention was given to the identification of calcium phosphate phases. Phase attribution was established from the presence of several characteristic reflections and their consistency with the overall diffraction pattern rather than from individual diffraction peaks alone. Microstructural observations were carried out using scanning electron microscopy coupled with energy-dispersive X-ray spectroscopy (SEM-EDS) (FEI Thermo Fisher XL30 FEG equipped with an X-MAX 150 mm^2^ silicon drift detector). Prior to analysis, specimens were sectioned using a precision low-speed saw, carbon-coated using a BALZERS CED 030 carbon evaporator, and subsequently rendered conductive by applying a silver-based conductive coating. SEM imaging was used to investigate pore morphology, pore distribution, and matrix homogeneity, whereas EDS analysis was employed to assess the spatial distribution of major elements within the geopolymeric matrices, elemental mapping and reaction products. The EDS results were used primarily for qualitative compositional assessment and elemental mapping should not be considered quantitative bulk chemical measurements.

## 3 Results and discussion

### 3.1 Characterization of precursors (VS and COS)

#### 3.1.1 Chemical composition of precursors

The chemical compositions of volcanic slag (VS) and calcined oyster shells (COS) are presented in Table S1. VS is primarily composed of SiO_2_ (40.89 wt%), Al_2_O_3_ (19.45 wt%) and Fe_2_O_3_ (16.69 wt%), with the combined content of these oxides exceeding 75 wt%. Such a composition satisfies the requirements generally considered favorable for geopolymer synthesis according to ASTM C618 and confirms the suitability of VS as an aluminosilicate precursor. Furthermore, the SiO_2_/Al_2_O_3_ molar ratio of 3.57 suggests an appropriate balance between silicate and aluminate species, promoting the formation of a highly cross-linked aluminosilicophosphate network during acid activation. In addition to its chemical composition, VS exhibits a specific surface area of 44 ± 1 m^2^ g^−1^, a micropore volume of 0.017 cm^3^ g^−1^, and a micropore surface area of 40.21 m^2^ g^−1^ determined by the *t*-plot method, with an average pore diameter of 0.55 nm (Table S2 and Fig. S3). These textural characteristics are expected to facilitate proton diffusion and enhance the dissolution of the reactive fraction of the precursor, thereby promoting the release of Si-, Al-, and Fe-containing species involved in geopolymer formation. By contrast, COS contains a high proportion of CaO (74.73 wt%), indicating an efficient conversion of CaCO_3_ during thermal treatment. The residual loss on ignition (23.23 wt%) is mainly associated with remaining carbonate species and secondary hydration or carbonation products formed after calcination. The marked compositional contrast between the aluminosilicate-rich VS and the calcium-rich COS is particularly relevant for phosphate-based geopolymerization because it promotes two parallel reaction pathways during acid activation: the formation of an aluminosilicophosphate gel and the precipitation of calcium phosphate phases. Consequently, the relative contribution of these pathways is expected to strongly influence pore generation, matrix development, and the final properties of the porous geopolymers. It should be noted that volcanic slag itself contains a significant amount of calcium (7.68 wt% CaO, Table S1). Therefore, calcium phosphate precipitation occurring during geopolymerization cannot be attributed exclusively to COS incorporation. Even in the reference formulations, calcium released from volcanic slag may contribute to the formation of calcium phosphate phases in the presence of phosphoric acid.

##### 3.1.1.1 Thermal behavior of precursors

The TGA/SDTA thermogram of oyster shells (Fig. 1a) exhibits three major thermal events. The first mass-loss region, accompanied by an endothermic peak centered at approximately 51 °C, is attributed to the removal of physically adsorbed water and the degradation of residual organic components present within the shell structure.^45^ Oyster shells consist of alternating inorganic and organic layers, in which proteins such as conchiolin act as structural binders between calcium carbonate crystallites.^44^ A second thermal event is observed around 468 °C and is assigned to the transformation of metastable aragonite into the thermodynamically more stable calcite polymorph, accompanied by the decomposition of residual organic matter trapped within the shell microstructure.^42,46^ This behavior is characteristic of biogenic calcium carbonate materials subjected to thermal treatment.

**Fig. 1:**
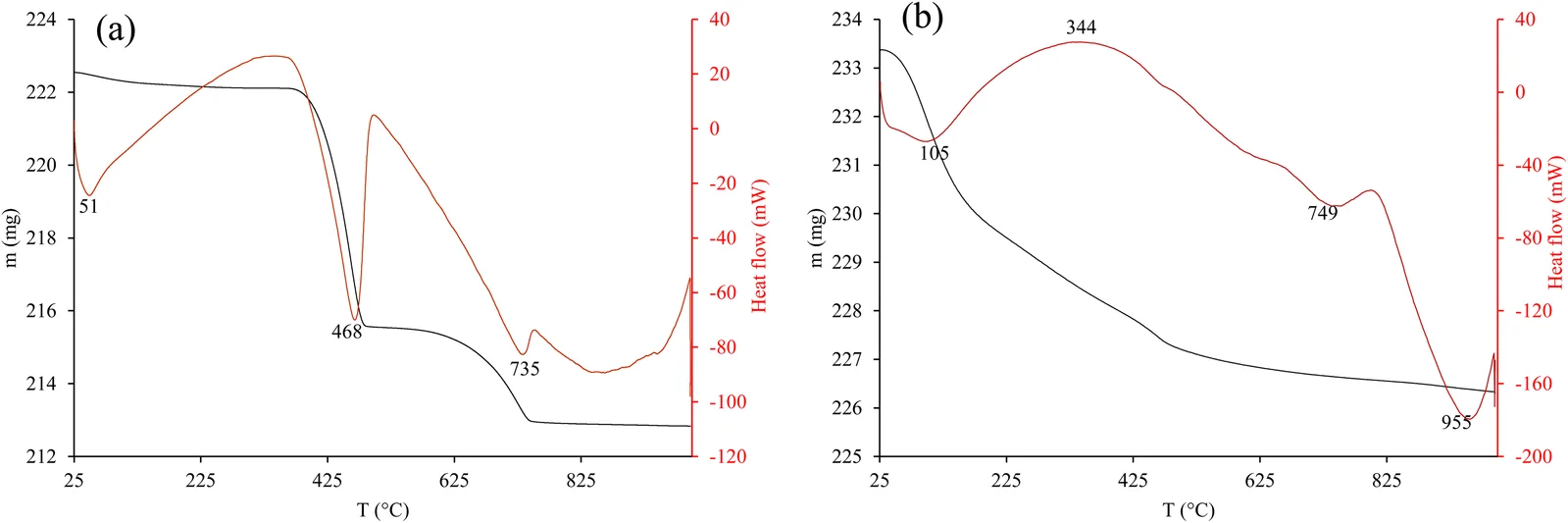
TGA (black curve)/SDTA (red curve) thermogram of OS (a) and VS (b).

The most pronounced thermal event occurs between 625 and 735 °C, and culminates in a strong endothermic peak at approximately 735 °C, which reflects the decomposition of CaCO_3_ into calcium oxide CaO and carbon dioxide CO_2_.^43^ Above 740 °C, the recorded mass remains nearly constant, indicating extensive decarbonation and confirming that calcination at 900 °C is sufficient to maximize the conversion of CaCO_3_ into calcium-rich phases available for subsequent geopolymerization reactions.

The thermal behavior of VS (Fig. 1b) differs markedly from that of oyster shells. A weak endothermic peak recorded at approximately 105 °C corresponds to the evaporation of physically adsorbed water, whereas the event observed near 344 °C is associated with the release of structurally bound water from hydrated mineral phases.^47^ Additional thermal events located at approximately 749 and 955 °C are attributed to crystallization and phase-reorganization processes occurring within the aluminosilicate framework during heating.^48,49^ The relatively low mass loss observed throughout the investigated temperature range confirms the high thermal stability of VS and its low organic matter content. Unlike kaolinitic clays, which require thermal activation to increase their reactivity, VS can be directly employed as a geopolymer precursor. More importantly, the contrasting thermal behaviors of VS and COS establish the basis for the subsequent development of porous geopolymers.

#### 3.1.2 Mineral composition of precursors

The XRD pattern of VS (Fig. 2) reveals the presence of several crystalline phases, including anorthite (CaAl_2_Si_2_O_8_), hedenbergite (CaFe(SiO_3_)_2_), albite (NaAlSi_3_O_8_), magnetite (Fe_3_O_4_), and alite (Ca_3_SiO_5_). These phases are fully consistent with the chemical composition determined by XRF (Table S1) and confirm the occurrence of silicate-, aluminosilicate-, calcium-, and iron-bearing minerals within the volcanic precursor.

**Fig. 2:**
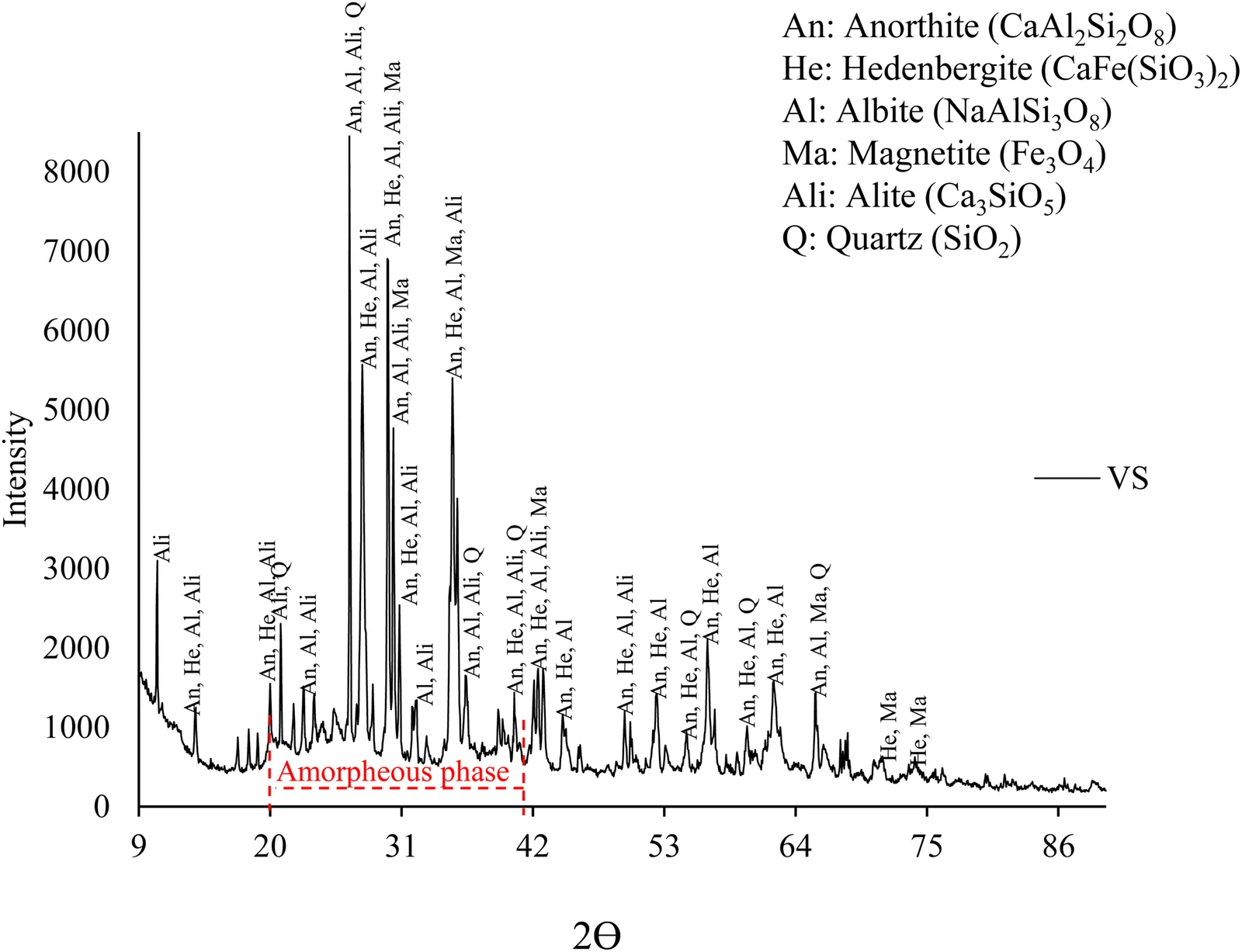
XRD diffractogram of VS.

In addition to these crystalline constituents, a broad diffuse halo extending from approximately 20 to 40° (2*θ*), is observed, indicating the presence of a significant amorphous fraction.^13,50^ Such poorly ordered aluminosilicate domains are generally considered more susceptible to acid dissolution than their crystalline counterparts and are therefore expected to contribute significantly to geopolymer formation. The coexistence of amorphous and crystalline phases is advantageous because it provides reactive species for gel formation while preserving relatively stable mineral phases that may contribute to the structural integrity of the final material.

The diffraction pattern of raw oyster shells (Fig. 3) confirms that aragonite is the dominant crystalline phase. Following calcination, most aragonite reflections disappear and the diffraction pattern becomes dominated by CaO-derived phases, particularly portlandite generated through the hydration of CaO upon exposure to atmospheric moisture, confirming the decarbonation process evidenced by thermal analysis.^39,42^ Residual calcite reflections remain detectable after calcination. Their persistence may result from incomplete decomposition of a fraction of the original CaCO_3_ or from partial recarbonation of CaO during storage. Furthermore, the broader diffraction peaks observed for COS indicate a reduction in crystallinity and crystallite size. This observation is consistent with particle-size estimates obtained from the Debye–Scherrer equation (Table S3) and SEM observations (Fig. S5D).

**Fig. 3:**
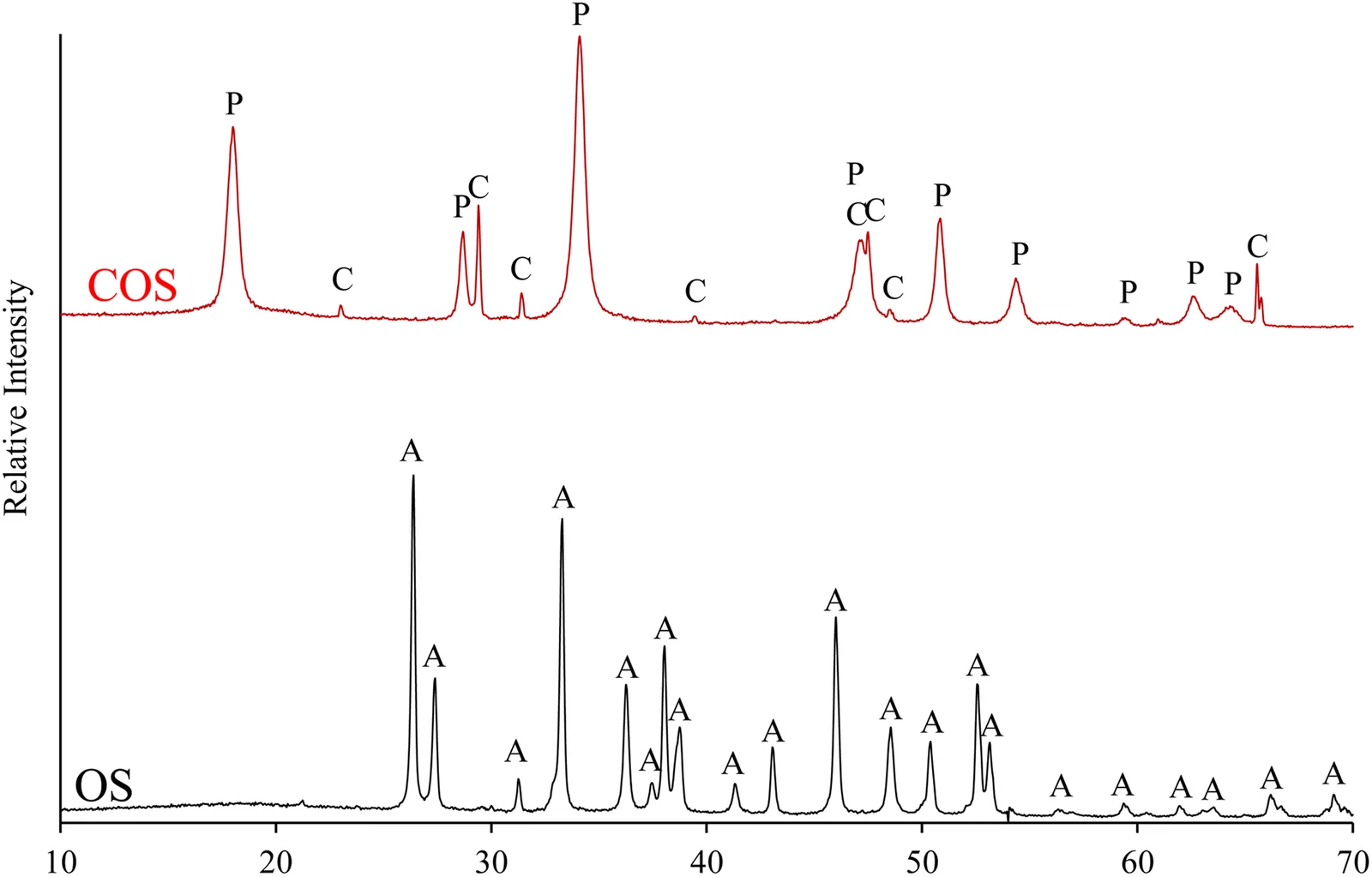
XRD diffractogram of OS (black curve) and COS (OS after calcination) (red curve).

Overall, the XRD results demonstrate that calcination effectively transforms oyster-shell-derived calcium carbonate into a readily available calcium source capable of participating in phosphate precipitation reactions. Combined with the reactive amorphous fraction identified in VS, this mineralogical assemblage establishes favorable conditions for the simultaneous formation of an aluminosilicophosphate gel and calcium phosphate phases during geopolymerization. The competition between these processes is expected to play a decisive role in matrix development, pore formation, and the resulting engineering properties.

##### 3.1.2.1 Overview of chemical bonds in precursors

The FTIR spectrum of VS (Fig. 4a) exhibits broad absorption bands centered at 3339 and 1629 cm^−1^, corresponding to the stretching and bending vibrations of molecular water, respectively.^50,51^ These bands are consistent with the dehydration events observed during TGA/SDTA analysis. The absorption band located at 963 cm^−1^ is assigned to the asymmetric stretching vibrations of Si–O–T bonds (T = Si, Al, or Fe), whereas the band at 514 cm^−1^ is attributed to bending vibrations involving Si–O–Al linkages, although contributions from Fe-containing aluminosilicate environments cannot be excluded.^13,14,52^ These spectral features confirm the aluminosilicate nature of VS and the presence of structural units capable of participating in dissolution and geopolymerization reactions.

**Fig. 4:**
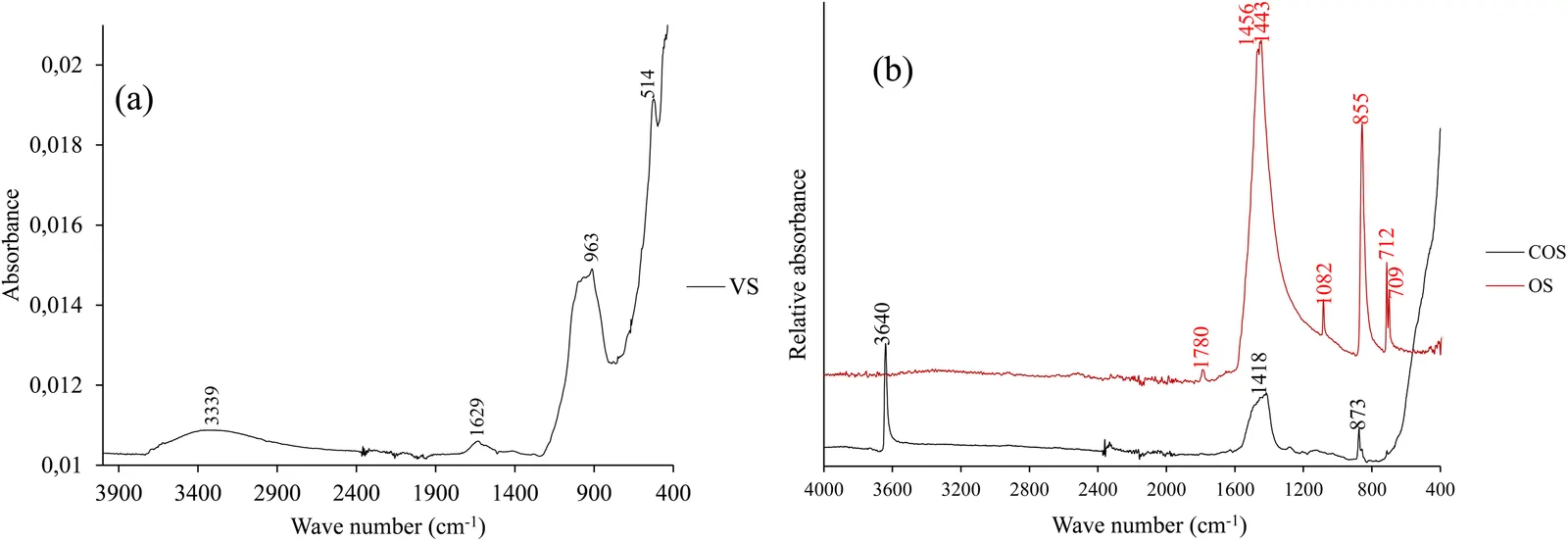
FT-IR spectrum of VS (a) and OS (b) before (red curve) and after calcination (black curve).

The FTIR spectrum of raw oyster shells (Fig. 4b) is dominated by bands characteristic of biogenic calcium carbonate. A weak absorption feature is observed near 1780 cm^−1^ and may be attributed to carbonyl-containing organic residues associated with the shell matrix; however, owing to its low intensity, this assignment should be treated with caution.^39^ The principal carbonate vibrations are observed at 1456 and 1443 cm^−1^ (asymmetric stretching vibration corresponding to the overlap of the carbonate with the organic residues), at 1082 and 855 cm^−1^ (out-of-plane deformation vibration) and finally at 712 and 709 cm^−1^ (in-plane deformation vibration) corresponding to the stretching and deformation modes of CO_3_^2−^ groups within the aragonite structure.^46,53,54^ Following calcination, the intensity of carbonate-related bands decreases substantially, confirming extensive decomposition of CaCO_3_. A new absorption band appears at approximately 3640 cm^−1^ and is assigned to O–H stretching vibrations associated with portlandite formed through the hydration of CaO under atmospheric conditions. Additional bands located at approximately 1418 and 873 cm^−1^ are characteristic of carbonate species and mainly indicate the presence of residual or secondary calcite formed through partial carbonation of CaO-derived phases.^42,55^

The excellent agreement between XRF, TGA/SDTA, XRD, and FTIR analyses confirms that VS provides a reactive aluminosilicate source containing both amorphous and crystalline phases, whereas COS acts as an effective calcium reservoir following the conversion of CaCO_3_ into CaO-derived phases.

### 3.2 Characterization of GVS_*x*_^*y*^COS porous geopolymers produced

#### 3.2.1 Macroscopic characteristics of the synthesized geopolymers

The visual appearance of the geopolymer specimens after 28 days of curing is shown in Fig. 5. Marked differences were observed as a function of both phosphoric acid concentration and COS content, demonstrating that these parameters strongly influenced the reaction extent and microstructural development. The GVS_10_^*y*^COS formulations exhibited a denser and more cohesive appearance than the corresponding GVS_6_^*y*^COS specimens. This behavior suggests a greater degree of precursor dissolution and a more extensive development of the aluminosilicophosphate binding matrix. The higher concentration of phosphoric acid provides a larger amount of reactive phosphate species and protons, thereby facilitating the dissolution of reactive aluminosilicate and calcium-bearing phases originating from VS and COS.

**Fig. 5:**
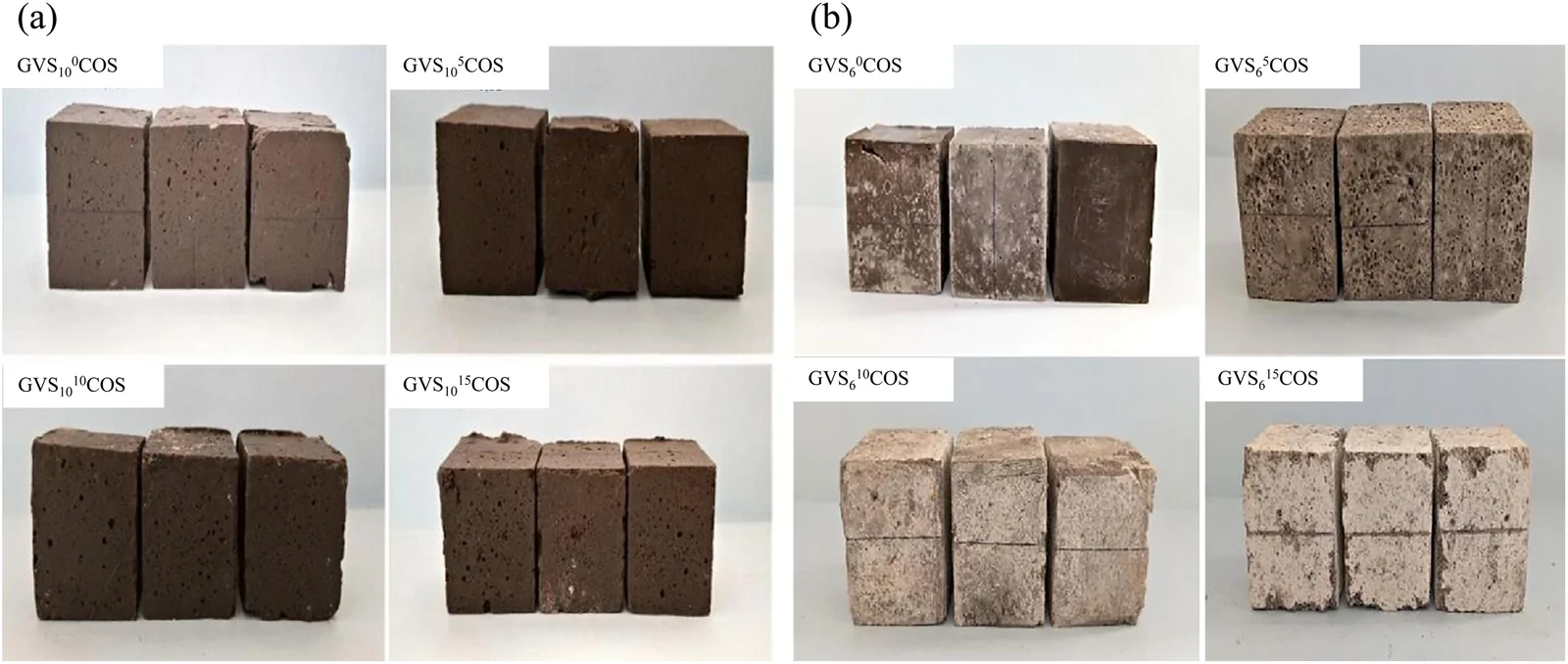
Geopolymer specimens activated by a phosphoric acid solution at 10 M (a) and 6 M (b) with different amounts of COS.

For both activation conditions, the progressive incorporation of COS increased visible pore formation. This observation indicates that calcium introduced by COS actively participates in the reaction process and modifies the evolution of the matrix during hardening. The development of visible porosity may be associated with the precipitation of calcium phosphate phases resulting from the interaction between dissolved calcium species and phosphate ions during acid activation. A possible mechanism is that the rapid precipitation of calcium phosphate reaction products locally modifies particle packing, consumes dissolved species, and generates heterogeneous regions within the reacting matrix, ultimately promoting the development of voids and pore-rich microdomains. A clear distinction was nevertheless observed between the two activation regimes. In the GVS_10_^*y*^COS system, pore formation occurred within a relatively compact matrix, suggesting that sufficient geopolymeric gel development was maintained despite the presence of calcium phosphate phases. Conversely, geopolymers activated with 6 mol L^−1^ phosphoric acid exhibited a less consolidated structure associated with increasing surface efflorescence as COS content increased. The occurrence of these white deposits indicates incomplete consumption of calcium-bearing species and reflects the lower dissolution capacity of the less acidic system. Under such conditions, unreacted calcium-containing phases may migrate toward the specimen surface during curing and subsequently react with atmospheric moisture and carbon dioxide, generating secondary carbonate products.^54,56^ The increase in efflorescence with COS incorporation further suggests that the additional calcium introduced into the system cannot be fully accommodated within the reaction products when precursor dissolution is limited. This observation provides the first macroscopic indication that the microstructure of these porous geopolymers is governed by the balance between geopolymeric network development and calcium phosphate precipitation. Such behavior is fully consistent with the deterioration of engineering properties observed at high COS contents and agrees with previous reports describing the adverse effects of efflorescence on geopolymer performance.^57^

#### 3.2.2 Microstructural analysis of the GVS_*x*_^*y*^COS (XRD)

The XRD patterns of the synthesized geopolymers are presented in Fig. 6 and 7. Compared with the raw volcanic slag, all geopolymer formulations exhibit a noticeable modification of the diffuse halo associated with the amorphous fraction of the precursor. Following phosphoric acid activation, this feature shifts toward higher diffraction angles, indicating substantial structural reorganization and the formation of a new amorphous aluminosilicophosphate phase. This displacement is more pronounced in GVS_10_^*y*^COS formulations than in GVS_6_^*y*^COS, suggesting a greater extent of precursor dissolution and reaction. However, because phosphoric acid concentration and L/S ratio varied simultaneously, this behavior should be interpreted as the combined effect of increased acid concentration and formulation-dependent differences in liquid availability. The increased proton availability promotes the breakdown of reactive aluminosilicate bonds and facilitates the release of soluble Si-, Al-, Fe-, and Ca-containing species that subsequently participate in gel formation.^5,58^ In contrast, the less pronounced modification of the diffuse halo in GVS_6_^*y*^COS formulations indicates incomplete precursor conversion and a lower degree of geopolymerization.

**Fig. 6:**
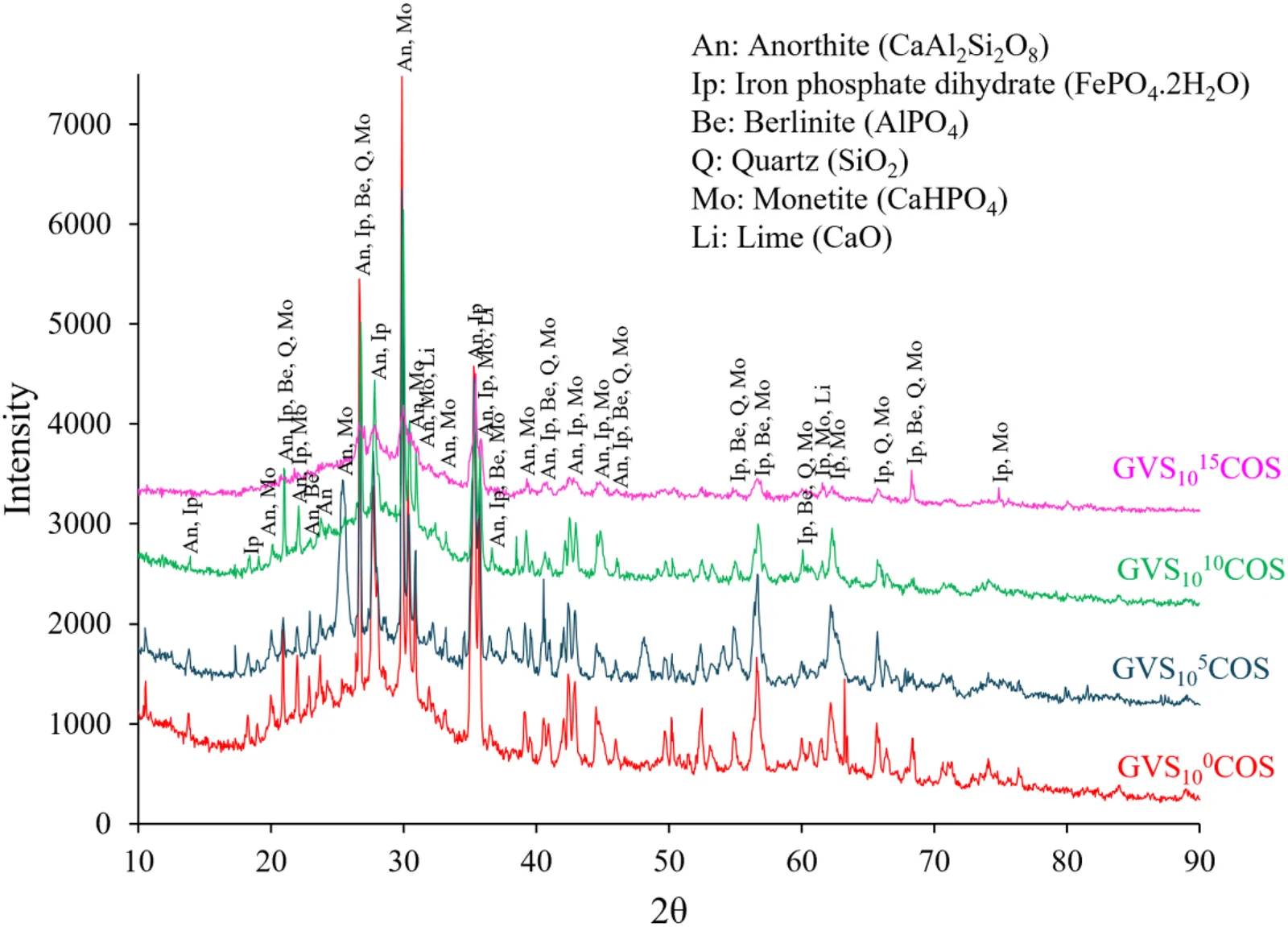
X-ray diffractograms of porous geopolymers at 10 mol L^−1^ of phosphoric acid as a function of %COS proportion.

**Fig. 7:**
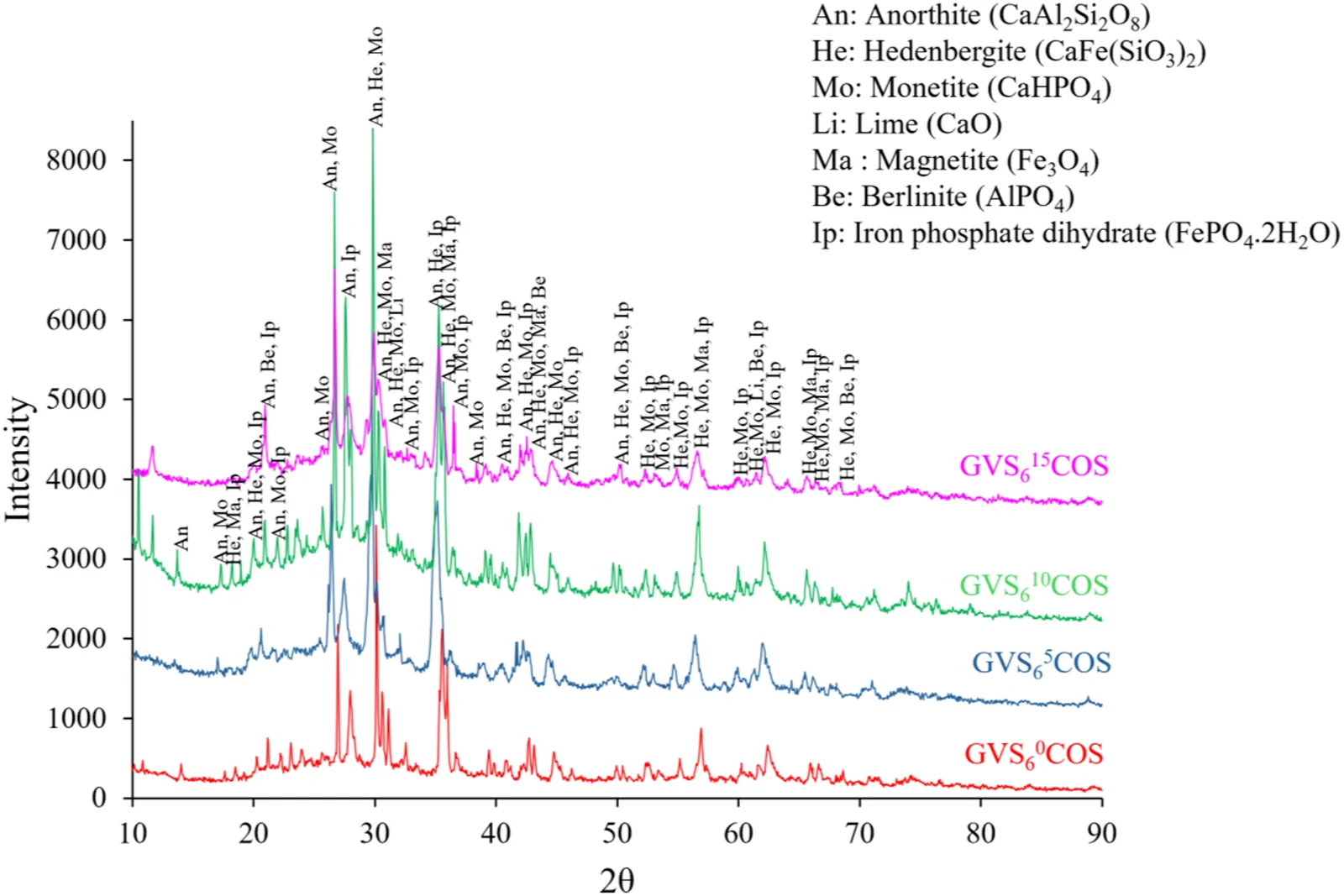
X-ray diffractograms of porous geopolymers at 6 mol L^−1^ of phosphoric acid as a function of %COS proportion.

Several crystalline phases inherited from the volcanic slag, particularly quartz and anorthite, remain detectable after curing, indicating their limited reactivity under the investigated conditions. By contrast, the persistence of hedenbergite and magnetite in GVS_6_^*y*^COS formulations, together with their disappearance or marked reduction in GVS_10_^*y*^COS systems, demonstrates that higher phosphoric acid concentration significantly enhances the dissolution of Fe-bearing and Ca-bearing minerals. This observation is consistent with the key role of Fe-containing phases in the hardening process of phosphate-based geopolymeric materials.^59^

Acid activation also led to the formation of new crystalline products, including berlinite (AlPO_4_), hydrated iron phosphate phases, and monetite (CaHPO_4_). The formation of berlinite indicates the reaction between dissolved aluminum species and phosphate ions, whereas monetite results from the precipitation of calcium released from both VS and COS.^20^ The presence of calcium-bearing phases in VS suggests that calcium phosphate formation may already occur in the reference formulations, whereas COS incorporation progressively increases the amount of calcium available for phosphate precipitation. The occurrence of monetite-related reflections in formulations containing little or no COS further supports the contribution of calcium initially present in VS. Therefore, the observed evolution of calcium phosphate phases should be interpreted as the result of increasing total calcium availability rather than as the exclusive consequence of oyster-shell-derived calcium.

Monetite identification was established from the simultaneous occurrence of several characteristic reflections assigned using Match! software and reference patterns from the Crystallography Open Database (COD). The assignment was based on the collective evolution of these reflections across the different formulations rather than on a single peak. Although some overlap with residual silicate and aluminosilicate phases inherited from volcanic slag cannot be completely excluded, the consistency of the diffraction patterns strongly supports the progressive formation of monetite with increasing COS incorporation. Because no Rietveld refinement or quantitative phase analysis was performed, the evolution of monetite reflections should be regarded as semi-quantitative evidence of increasing calcium phosphate precipitation rather than as a direct measurement of phase fraction.

The coexistence of amorphous aluminosilicophosphate gel and crystalline calcium phosphate phases reveals the presence of competing reaction pathways during geopolymerization. At moderate COS contents, these processes appear to evolve simultaneously, allowing the formation of a cohesive porous matrix. However, as COS incorporation increases, calcium phosphate precipitation becomes increasingly significant. Under these conditions, a greater proportion of reaction products is associated with calcium phosphate phases, which may contribute to limiting the development of a continuous geopolymeric network. This interpretation is supported by the progressive increase in monetite reflections, and the microstructural evolution observed by SEM. Although the formation of monetite is clearly evidenced by XRD, precipitation alone does not necessarily generate pores. Instead, it is proposed that the rapid formation of calcium phosphate phases modifies local reaction pathways, disrupts homogeneous gel development, and creates heterogeneous packing conditions during hardening. These effects may favor the formation of void spaces within the microstructure.

The detection of residual calcium-containing phases and carbonation products in the low-acidity formulations further supports this interpretation and is consistent with the pronounced efflorescence observed macroscopically. Consequently, the XRD results suggest that the observed microstructural evolution and apparent pore development may be associated with the relative contributions of aluminosilicophosphate gel formation and calcium phosphate precipitation. However, quantitative porosity measurements would be required to establish a direct causal relationship. Compared with previous studies employing conventional calcium-rich additives, the present results provide additional insight into the evolution of calcium phosphate phases in volcanic-slag-based phosphate geopolymers containing oyster-shell-derived calcium.

#### 3.2.3 Study of chemical bonds of GVS_*x*_^*y*^COS

The FTIR spectra of the synthesized geopolymers are shown in Fig. 8. Regardless of formulation, broad absorption bands located near 3300 and 1631 cm^−1^ remain visible after curing, indicating the presence of residual water associated with hydrated phosphate phases and the amorphous geopolymeric matrix. The most significant spectral modification relative to the precursor is the appearance of strong absorption bands centered around 1030 and 915 cm^−1^. These bands reflect major structural rearrangements induced by phosphoric acid activation and are attributed to phosphate-containing environments involving Si–O–P, Al–O–P, and P–O bonds within the newly formed aluminosilicophosphate framework.^13,23,39,60^ The emergence of these bands confirms the incorporation of phosphate species into the reaction products formed during acid activation. Their development is consistent with the coexistence of amorphous aluminosilicophosphate gel and phosphate-containing crystalline phases identified by XRD, including berlinite, hydrated iron phosphates, and monetite. Therefore, the FTIR results corroborate the phase assemblages revealed by diffraction analysis and confirm that phosphate species participate in both amorphous and crystalline reaction products. A slight displacement of the band initially located at 514 cm^−1^ in VS toward approximately 519–521 cm^−1^ is also observed after geopolymerization. This shift is consistent with structural modifications associated with the formation of Al-containing phosphate environments and supports the presence of berlinite identified by XRD.^54^

**Fig. 8:**
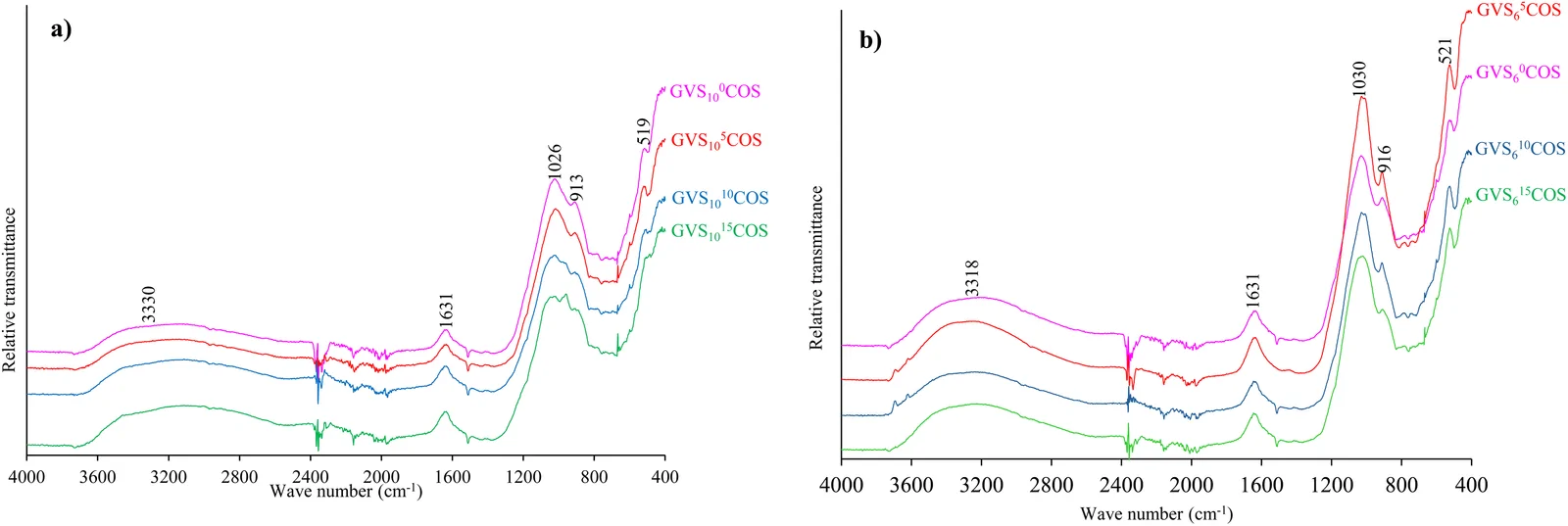
FT-IR spectra of geopolymers with activating phosphoric acid solution of 10 M (a) and 6 M (b).

Clear differences are observed between the two activation conditions. The broader and more intense phosphate-related absorptions observed in GVS_10_^*y*^COS formulations suggest a higher degree of structural reorganization and precursor conversion. Nevertheless, these differences likely reflect the combined influence of phosphoric acid concentration and the corresponding variation in L/S ratio. By contrast, the narrower bands observed in GVS_6_^*y*^COS specimens suggest a lower reaction extent and are consistent with the presence of residual precursor phases detected by XRD.

Furthermore, the higher degree of structural reorganization evidenced by the broader and more intense phosphate-related bands in GVS_10_^*y*^COS formulations is fully consistent with the denser and more homogeneous microstructures observed by SEM. Collectively, the FTIR and XRD results indicate that phosphoric acid concentration strongly influences the balance between aluminosilicophosphate gel formation and calcium phosphate precipitation, thereby governing the final microstructure of the geopolymers.

#### 3.2.4 Microstructural evolution of porous geopolymers

Representative SEM micrographs (Fig. 9) reveal a strong influence of both COS content and phosphoric acid concentration on microstructural development. The reference geopolymers exhibited relatively dense and homogeneous matrices with limited pore formation, indicating that the calcium naturally present in VS already participates in phosphate precipitation reactions. Consequently, COS should be regarded as an additional calcium source that progressively increases calcium availability rather than the sole origin of calcium phosphate formation.

**Fig. 9:**
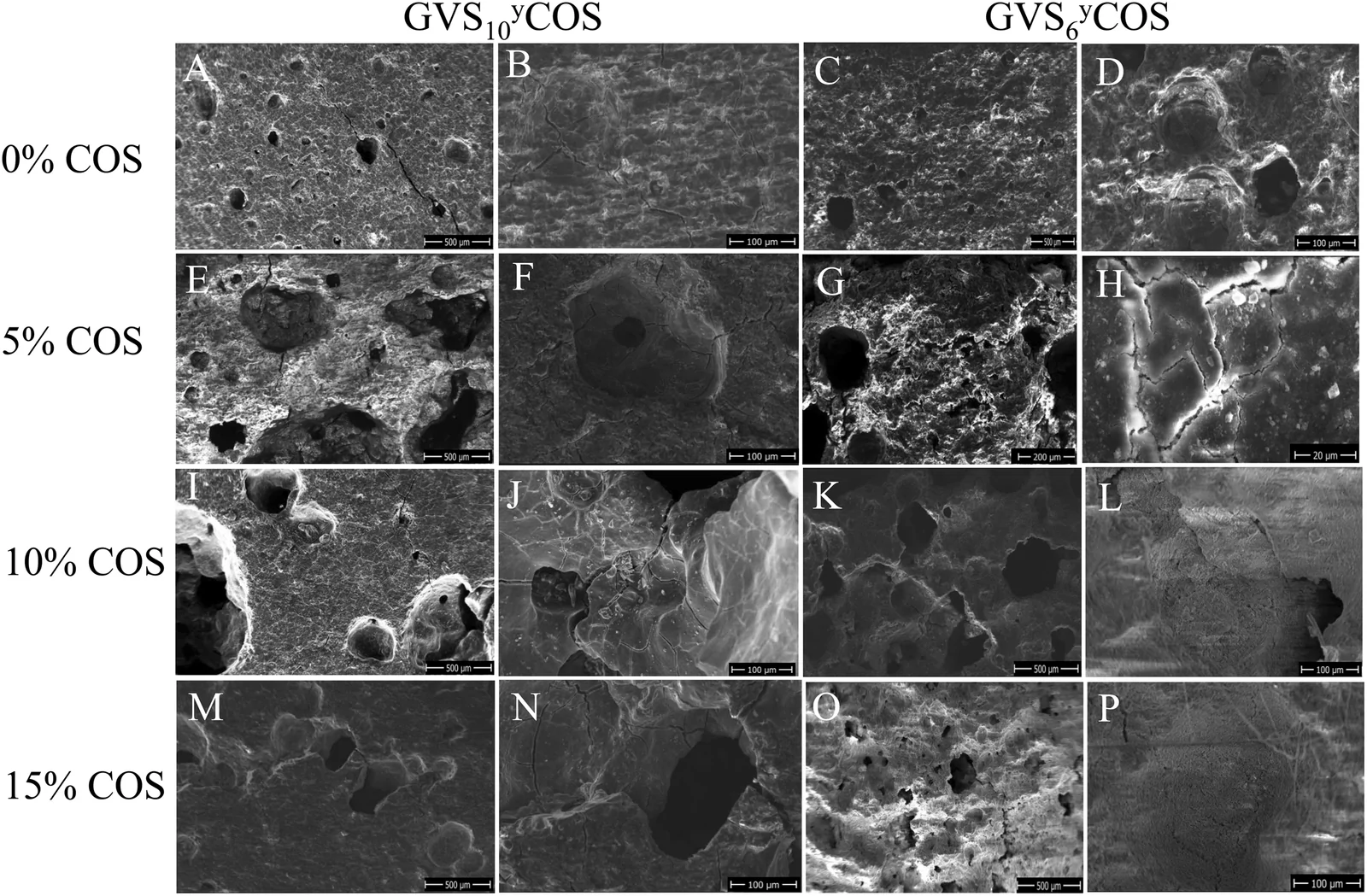
SEM images of porous geopolymers GVS_10_^0^COS (A and B), GVS_10_^5^COS (E and F), GVS_10_^10^COS (I and J), GVS_10_^15^COS (M and N), GVS_6_^0^COS (C and D), GVS_6_^5^COS (G and H), GVS_6_^10^COS (K and L), GVS_6_^15^COS (O and P).

COS incorporation promoted the formation of a hierarchical pore structure composed of interconnected macro- and secondary pores. At 5 wt% COS, porosity remained homogeneously distributed within a continuous matrix, suggesting a favorable balance between aluminosilicophosphate gel formation and calcium phosphate precipitation. In contrast, higher COS contents led to increasing microstructural heterogeneity, particularly in GVS_6_^*y*^COS formulations, where partially reacted particles indicate incomplete dissolution of volcanic slag and calcium-rich phases.

These poorly consolidated regions are consistent with the higher water absorption, stronger efflorescence, and lower compressive strength observed for the corresponding specimens. Conversely, the more homogeneous microstructure observed in GVS_10_^*y*^COS geopolymers is consistent with enhanced precursor dissolution and improved matrix development. However, this behavior may result from the combined influence of higher phosphoric acid concentration and formulation-dependent L/S ratio. EDS analyses (Fig. S6 and S7) qualitatively confirmed the presence and spatial distribution of Si, Al, Fe, P, O, and Ca, within the investigated samples, supporting the formation of phosphate-containing reaction products identified by XRD and FTIR. However, the EDS maps were used primarily for qualitative visualization of elemental distribution and should not be interpreted as quantitative bulk compositional evidence.

Overall, the combined microstructural and spectroscopic results indicate that the final pore structure is governed by the balance between matrix development through aluminosilicophosphate gel formation and pore generation associated with calcium phosphate precipitation. Although SEM observations clearly reveal the presence of pores and microstructural modifications induced by COS incorporation, the present study does not include direct quantitative porosity measurements. Therefore, the proposed contribution of calcium phosphate precipitation to pore formation should be regarded as a supported interpretation rather than definitive proof. Based on the combined XRD and SEM observations, a plausible pore-development mechanism can be proposed. During phosphoric acid activation, calcium released from both VS and COS rapidly reacts with phosphate species to form calcium phosphate phases, predominantly monetite. The precipitation of these phases may locally compete with aluminosilicophosphate gel formation, resulting in heterogeneous particle packing and uneven matrix densification. As the matrix hardens, these heterogeneities may promote the formation of voids and pore-rich regions. Therefore, pore development is proposed to result from indirect microstructural effects associated with calcium phosphate precipitation rather than from precipitation itself.

### 3.3 Mechanical characterization of porous geopolymers

#### 3.3.1 Setting time

The setting times of the fresh geopolymer pastes are presented in Fig. 10, and the corresponding values are reported in Table S3. The evolution of setting time was influenced by both COS incorporation and the associated increase in L/S ratio. Therefore, the observed acceleration of setting reflects the combined effect of calcium availability and formulation-dependent changes in fresh paste composition. A marked reduction in setting time was observed following the addition of 5 wt% COS, irrespective of the phosphoric acid concentration.

**Fig. 10:**
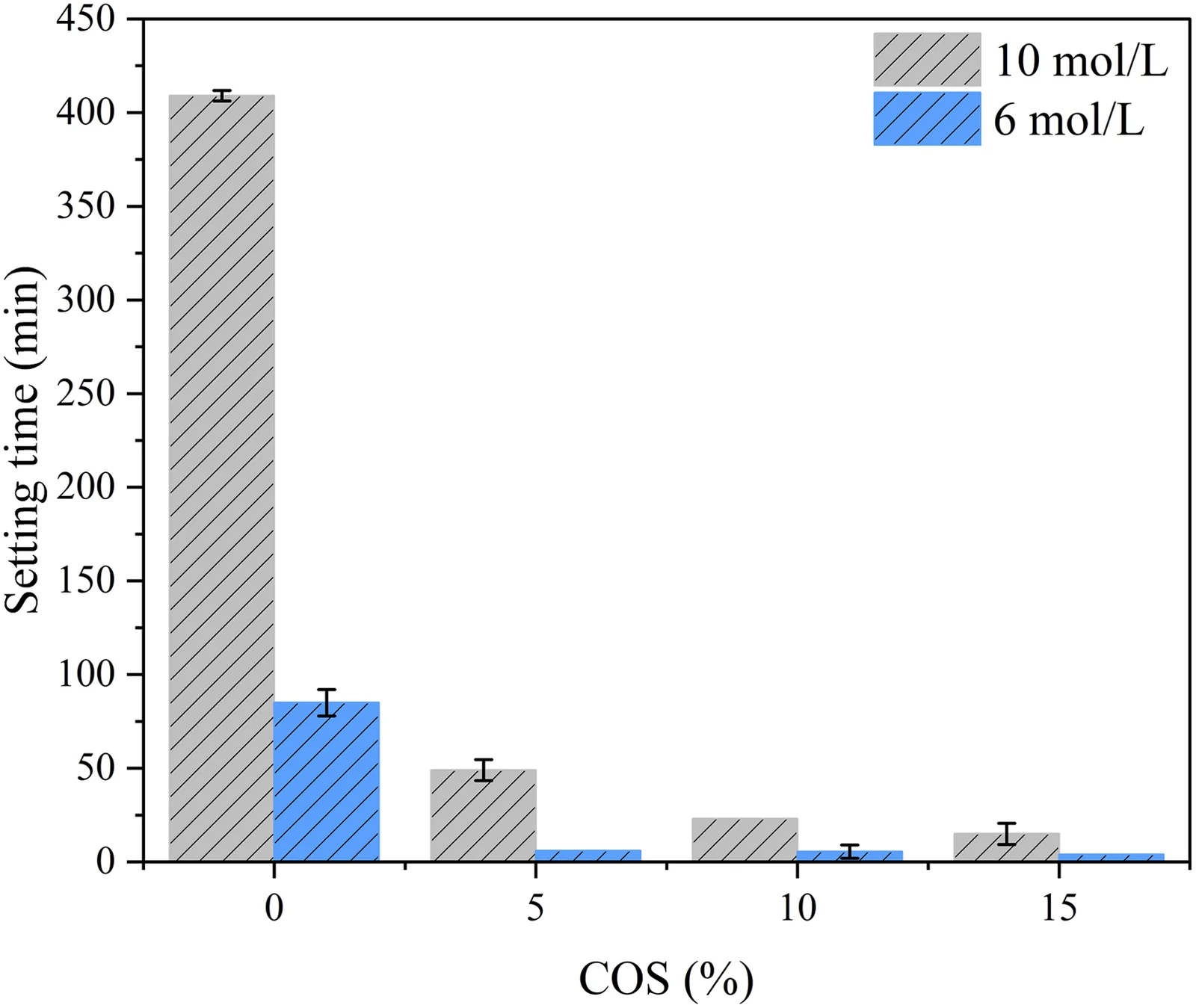
Setting time of geopolymers.

This behavior is attributed to the rapid interaction between dissolved calcium species and phosphate ions, resulting in the precipitation of calcium phosphate phases. Compared with the progressive polycondensation of the aluminosilicophosphate gel, calcium phosphate precipitation occurs at a faster rate and contributes to the early rigidification of the matrix.^61^ The formation of these poorly soluble phases reduces the mobility of dissolved species within the fresh paste and accelerates the transition from a fluid suspension to a consolidated material. This interpretation is consistent with the progressive increase in monetite reflections identified by XRD and the increasing abundance of calcium-containing reaction products revealed by the microstructural analyses.

A substantial difference in setting time was observed between GVS_10_^0^COS and GVS_6_^0^COS. This behavior reflects the combined influence of phosphoric acid concentration and liquid-to-solid ratio (Table 1). Therefore, the faster setting behavior observed in one formulation series cannot be attributed exclusively to phosphoric acid concentration. Increasing both parameters enhances proton availability and promotes the dissolution of reactive aluminosilicate and calcium-bearing phases. Consequently, larger quantities of reactive species remain available in solution prior to precipitation and polycondensation, resulting in longer setting times. These observations are consistent with the greater structural reorganization evidenced by XRD and FTIR for geopolymers activated with 10 mol L^−1^ phosphoric acid and agree with previous findings reported for phosphate-based geopolymer systems.^6,17,59^

#### 3.3.2 Water absorption

The water absorption values measured after 28 days of curing are presented in Fig. 11 and summarized in Table S3. The evolution of this parameter strongly depended on both phosphoric acid concentration and COS incorporation, reflecting significant differences in pore architecture and matrix continuity. For GVS_6_^*y*^COS formulations, water absorption increased progressively with COS content, reaching its highest value at 15 wt% COS. This behavior is consistent with the SEM observations, which revealed increasing microstructural heterogeneity and a greater abundance of partially reacted particles at high COS contents.

**Fig. 11:**
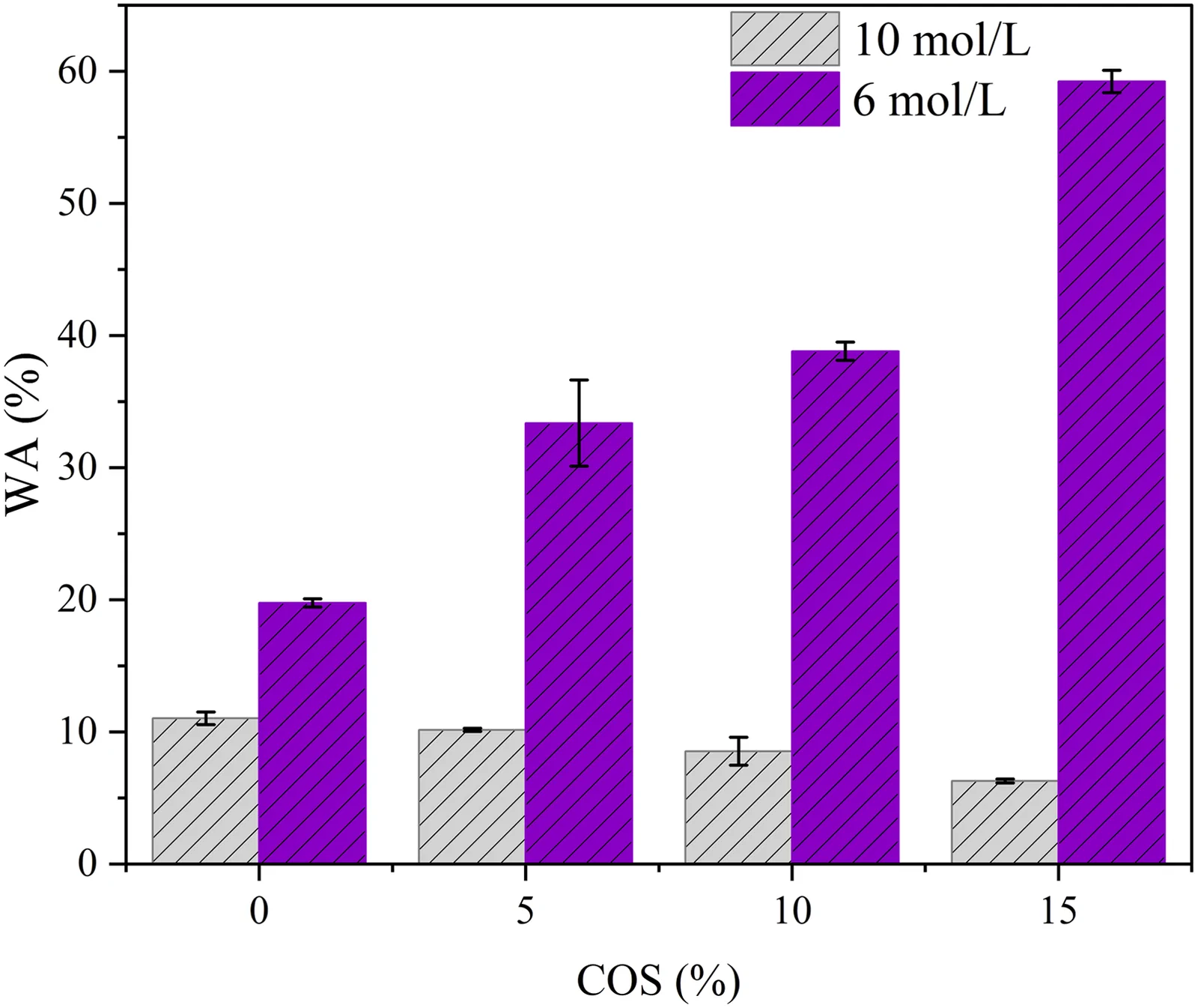
Water absorption of geopolymers.

The incomplete dissolution of VS and calcium-rich phases generated poorly consolidated regions and interconnected capillary pathways that facilitated water penetration throughout the matrix. In contrast, GVS_10_^*y*^COS geopolymers exhibited significantly lower water absorption despite the increase in apparent macroporosity observed by SEM. This apparent contradiction suggests that water transport may be influenced not only by pore volume but also by factors such as pore size distribution, pore-network connectivity, and matrix continuity. However, these parameters were not measured directly in the present study and therefore remain plausible interpretations of the observed behavior.^12,14,62^

Although COS incorporation promoted the formation of additional macropores, the higher phosphoric acid concentration simultaneously enhanced the extent of precursor dissolution and favored the development of a denser aluminosilicophosphate matrix, as evidenced by XRD, FTIR, and SEM analyses.

The decrease in water absorption despite the increase in apparent macroporosity suggests that the newly generated pores contributed only marginally to capillary water uptake. Instead, the observed water absorption behavior may be related to differences in pore-network connectivity and matrix continuity. Nevertheless, direct pore-network characterization would be required to confirm this interpretation.

Therefore, the low water absorption observed in these formulations is consistent with greater matrix continuity and densification resulting from more extensive geopolymerization, which may outweigh the influence of increased visible porosity. This interpretation is further supported by the higher compressive strengths measured for GVS_10_^*y*^COS formulations, confirming the formation of a more continuous and less permeable microstructure.

These results indicate that pore development and matrix formation occurred simultaneously during geopolymerization. Although pore development occurred concomitantly with calcium phosphate precipitation, enhanced precursor dissolution favored the formation of a continuous binding matrix. Consequently, water absorption trends observed in this study are consistent with a balance between pore-network organization and matrix densification, although the exact contribution of pore connectivity could not be quantified.

The increase in visible pore development observed by SEM correlates with the evolution of water absorption. Nevertheless, water absorption cannot be considered a direct quantitative measure of total porosity. Therefore, the relationship between calcium phosphate precipitation and pore development remains inferential. Similar relationships between pore structure, matrix development, and absorption behavior have been reported for porous geopolymer systems.^21,25^ It should nevertheless be recognized that the progressive increase in L/S ratio accompanying COS incorporation may also contribute to the observed changes in water absorption. Therefore, the present results do not allow complete separation of COS effects from those associated with formulation-dependent L/S variations.

#### 3.3.3 Studies of dimensional variations of the porous geopolymers (linear shrinkage)

The linear shrinkage values measured after 28 days of curing are presented in Fig. 12 and reported in Table S3. Distinct behaviors were observed depending on the activation conditions. For GVS_10_^*y*^COS formulations, shrinkage progressively decreased with increasing COS content, whereas a slight increase was observed for GVS_6_^*y*^COS specimens. These differences can be directly related to the reaction extent and microstructural evolution identified in the previous sections. The higher phosphoric acid concentration promoted a greater extent of precursor dissolution and more extensive development of the aluminosilicophosphate gel. During drying, the removal of physically bound water generated capillary stresses within the pore network, contributing to dimensional contraction.^63,64^ Simultaneously, COS incorporation promoted pore development associated with calcium phosphate formation. In highly reacted systems such as GVS_10_^*y*^COS, the additional pore volume likely provided void space capable of partially accommodating these drying stresses, thereby contributing to shrinkage reduction. The behavior of GVS_6_^*y*^COS specimens differed because their lower reaction extent resulted in the coexistence of reaction products and significant quantities of partially reacted particles. Under such conditions, the formation of efflorescence and secondary carbonation products generated localized expansion phenomena that partially counterbalanced the normal shrinkage associated with drying. This interpretation is consistent with the visual observation of efflorescence and with the presence of residual calcium-containing phases detected by XRD. Despite these differences, all formulations exhibited shrinkage values below 2%, indicating satisfactory dimensional stability. These findings demonstrate that COS can promote pore development without inducing excessive volumetric instability, unlike highly reactive foaming agents such as hydrogen peroxide.^6^

**Fig. 12:**
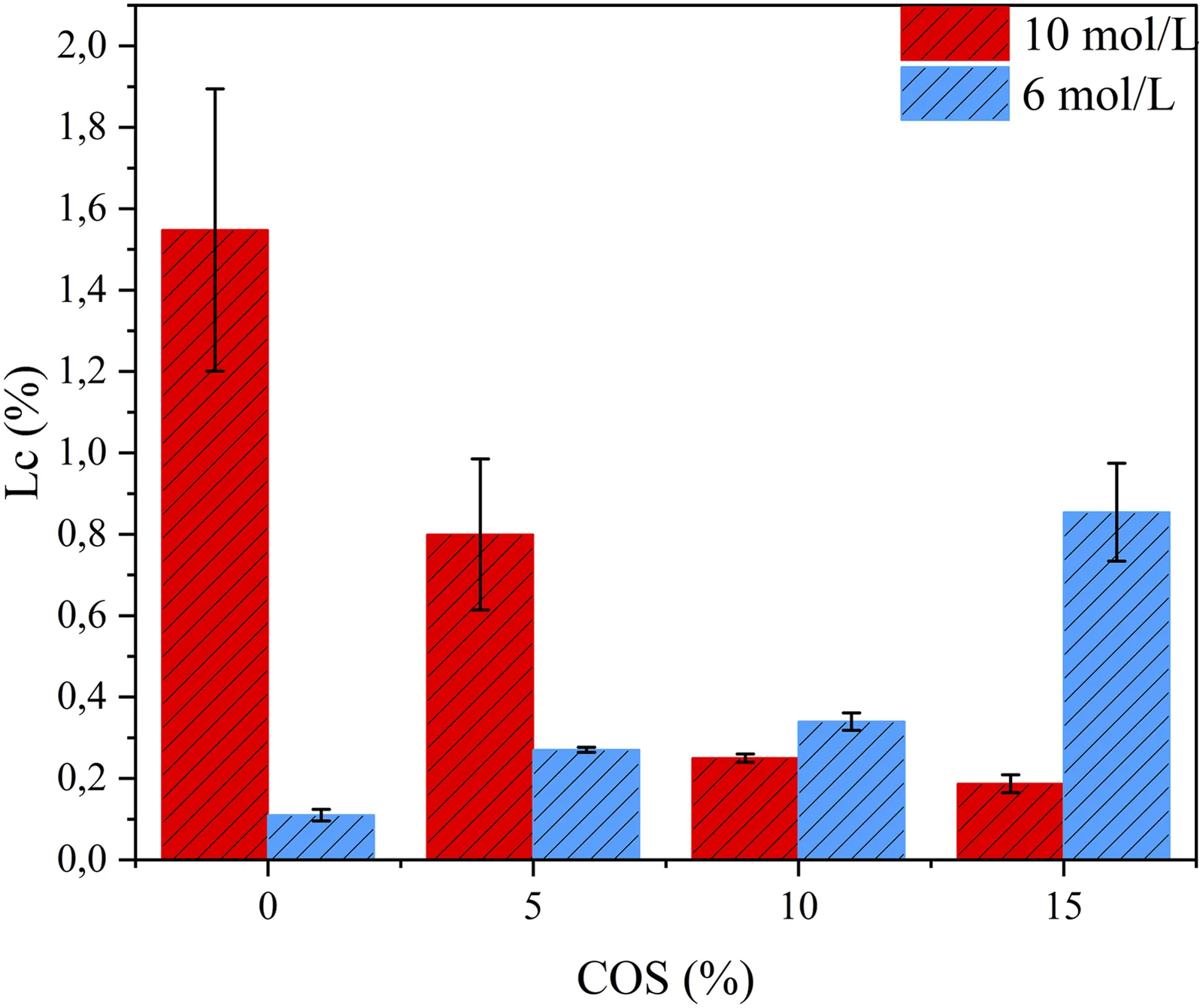
Linear shrinkage on drying of different geopolymers during the 28 days drying.

#### 3.3.4 Compressive strength

The compressive strengths of the geopolymer specimens after 28 days of curing are presented in Fig. 13 and summarized in Table S3. The mechanical performance was influenced by phosphoric acid concentration, COS incorporation, and the corresponding increase in L/S ratio. Therefore, the observed strength evolution should be interpreted as the combined effect of these parameters rather than the sole consequence of COS addition.

**Fig. 13:**
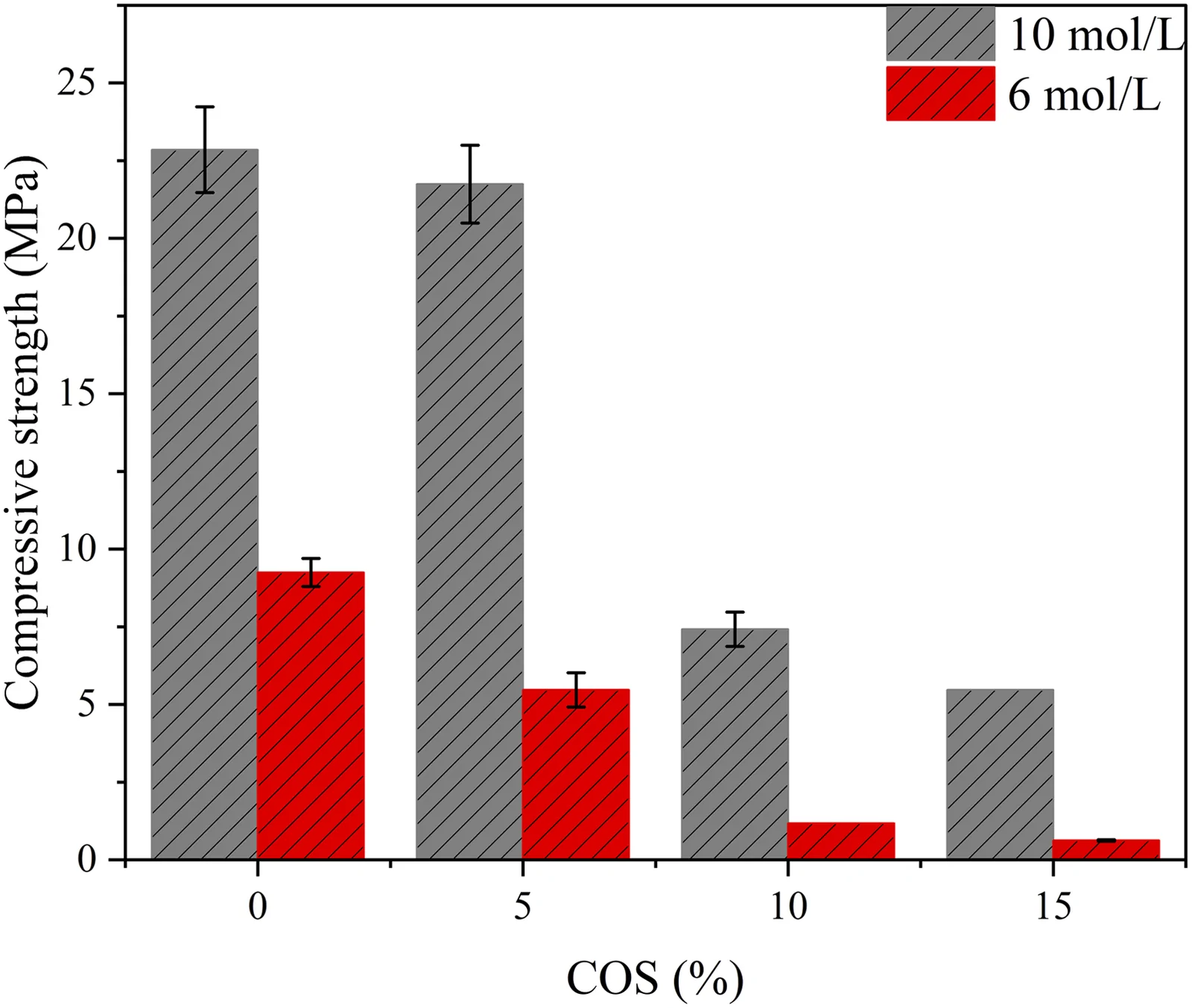
Compressive strength of geopolymers aged 28 days.

The reference formulations exhibited the highest compressive strengths within each activation system, with GVS_10_^0^COS significantly outperforming GVS_6_^0^COS. This difference is consistent with a greater extent of precursor dissolution achieved at 10 mol L^−1^ phosphoric acid, which promoted the formation of a more continuous and homogeneous aluminosilicophosphate matrix. However, because phosphoric acid concentration and L/S ratio varied simultaneously, the improved mechanical performance should be interpreted as the combined effect of these parameters rather than the consequence of acid concentration alone. The larger displacement of the amorphous halo observed by XRD, the stronger phosphate-related FTIR absorptions, and the denser microstructures revealed by SEM collectively support this interpretation. Enhanced dissolution facilitates the release of reactive species required for phosphate-containing reaction products and promotes the development of a cohesive binding matrix.^14,59,64^ In addition, the higher liquid-to-solid ratio likely improved particle dispersion and matrix consolidation during curing, resulting in superior mechanical performance. Similar trends have been reported for other geopolymer systems.^65^ The incorporation of 5 wt% COS preserved most of the original compressive strength while promoting the development of a homogeneous porous microstructure associated with calcium phosphate precipitation reactions. SEM observations revealed that pores remained relatively well distributed within a continuous matrix, indicating that aluminosilicophosphate gel formation remained sufficiently extensive to ensure efficient stress transfer throughout the material. Under these conditions, the generated porosity did not significantly compromise structural integrity because the continuous aluminosilicophosphate matrix remained capable of effectively transferring applied stresses. Consequently, the 5 wt% COS formulations achieved the most favorable balance between pore development and matrix cohesion.

Beyond 5 wt% COS, compressive strength decreased markedly for both activation conditions. This reduction cannot be attributed solely to an increase in porosity but rather to the simultaneous evolution of pore structure and matrix continuity. XRD results revealed increasing monetite formation, whereas SEM observations showed the progressive accumulation of partially reacted particles and poorly consolidated regions. These observations suggest that calcium phosphate precipitation progressively increased relative to aluminosilicophosphate gel development and may contribute to a more heterogeneous microstructure.^20,21^ The observed increase in apparent pore volume development and microstructural discontinuities may have reduced the effective load-bearing cross-section and promoted stress concentration under compressive loading. Simultaneously, the reduced continuity of the aluminosilicophosphate matrix limited efficient stress redistribution, thereby facilitating crack initiation and propagation. This interpretation is consistent with the increase in water absorption, the occurrence of efflorescence, and the microstructural heterogeneity observed for the corresponding formulations.

The strong correlation between phase assemblage, pore structure, and compressive strength suggests that the mechanical performance of the synthesized geopolymers is primarily governed by phase assemblage and microstructural evolution, which reflect the balance between matrix densification and pore development. While increased apparent pore development appears to be associated with calcium phosphate precipitation, the available results suggest that excessive precipitation may locally disrupt matrix continuity and contribute to a less homogeneous microstructure. Consequently, the superior performance of the 5 wt% COS formulations suggests that, among the compositions investigated, this formulation provided the most favorable balance between pore development and matrix continuity.

### 3.4 Environmental implications

Although the developed porous geopolymers exhibited promising microstructural and mechanical properties, their potential large-scale implementation must be assessed from both economic and application-oriented perspectives. Phosphate-based geopolymer systems intrinsically differ from conventional alkali-activated materials owing to the relatively high cost of phosphoric acid, which constitutes the main cost contributor in the present formulations. Consequently, the economic competitiveness of these materials for conventional structural applications may be limited when compared with Portland cement and alkali-activated binders.

However, the objective of the present work was not to develop a structural construction material, but rather to investigate the feasibility of producing porous functional geopolymers from locally available resources and industrial wastes and to contribute to the understanding of how biogenic calcium-rich residues influence reaction pathways and microstructural development in phosphate-based geopolymer systems. In this context, the use of volcanic slag requires no thermal activation, thereby reducing energy consumption during precursor processing. Furthermore, oyster shells are an abundant biogenic waste stream that is generally discarded without valorization. Their conversion into calcined oyster shells offers a potential route for waste recovery and the production of value-added materials. However, the overall environmental benefit remains to be quantitatively verified through dedicated life-cycle and techno-economic assessments (LCA).

Commercial foaming agents commonly used for porous geopolymers, such as hydrogen peroxide, metallic aluminum powder, and peracetic acid, represent an additional production cost and may present handling or safety constraints. By contrast, COS acts simultaneously as a waste-derived calcium source and a pore-development promoter through calcium phosphate formation, reducing the need for dedicated foaming additives. Among the investigated formulations, the composition containing 5 wt% COS was particularly attractive because it generated a homogeneous porous microstructure while preserving most of the original compressive strength, thereby maximizing the functional benefit obtained from a relatively small amount of waste-derived additive.

The economic relevance of phosphate-based porous geopolymers is therefore expected to depend on the targeted application. For conventional structural applications, the use of phosphoric acid may not be economically justified. Conversely, for applications that may benefit from controlled porosity, chemical stability, and low-density materials, such as filtration media, adsorbents, catalyst supports, and specialized environmental remediation systems, material functionality rather than compressive strength may become the primary performance criterion. No filtration tests, adsorption measurements, permeability evaluations, catalytic activity assessments, or long-term stability studies were performed in the present work. Therefore, these applications should be regarded as potential future directions rather than demonstrated end uses. Under these conditions, the valorization of locally available volcanic resources and oyster-shell waste may partially offset the cost of the activating solution while offering a promising waste-valorization pathway.

Nevertheless, a comprehensive techno-economic assessment and LCA were beyond the scope of the present study. Consequently, the environmental performance of the proposed materials cannot presently be quantified. Future work should therefore include detailed LCA, cost modeling, optimization of phosphoric acid consumption, and evaluation of alternative phosphate sources. In addition, the long-term durability of porous phosphate geopolymers remains insufficiently documented and represents one of the principal limitations of the present study. Future investigations should evaluate prolonged water exposure, wet-dry cycling, chemical attack, and leaching behavior and long-term microstructural stability under realistic service conditions before practical implementation can be considered.

## 4 Conclusion

The present work contributes to the growing body of research on phosphate-based geopolymers by demonstrating the feasibility of combining volcanic slag (VS) and calcined oyster shells (COS) waste as a calcium-rich bio-derived additive with a single porous material production route. Beyond the use of waste-derived calcium source, the study provides insight into the relationship between calcium availability, calcium phosphate formation, microstructural evolution and engineering performance in phosphoric-acid activated systems. The results suggest that the reaction process was influenced by the balance between aluminosilicophosphate matrix formation and calcium phosphate precipitation. Increasing phosphoric acid concentration was associated with enhanced precursor dissolution, greater matrix continuity, and improved engineering performance. However, because differences in phosphoric acid concentration were accompanied by variations in liquid-to-solid ratio, these effects should be interpreted as the combined influence of both parameters. XRD, FTIR, and SEM analyses consistently revealed that COS incorporation modified phase assemblage and microstructural evolution through the formation of calcium phosphate phases, which may contribute indirectly to pore development by altering matrix organization and densification during hardening. The results also indicate that VS itself contributes calcium to the system and therefore participates in phosphate precipitation reactions even in the absence of COS. However, because the present analyses are primarily qualitative or semi-quantitative, additional quantitative phase analysis and chemical characterization would further strengthen the interpretation of reaction pathways and phase evolution. Among the investigated formulations and corresponding L/S, the 5 wt% COS mixture provided the most favorable compromise between porosity and compressive strength. However, this result should not be interpreted as a universal optimum, since only four COS contents (0, 5, 10, and 15 wt%) were evaluated. Intermediate compositions may potentially yield different performance balances and deserve further investigation. While moderate COS incorporation generated a homogeneous pore structure without significantly disrupting matrix continuity, higher COS contents were associated with increased microstructural heterogeneity, more pronounced efflorescence, and lower mechanical performance. The results further suggest that engineering properties were controlled not only by the amount of porosity, but also potentially by pore-network connectivity and matrix continuity. However, quantitative characterization of the pore network would be required to confirm these relationships. From a recovery perspective, the proposed approach offers a promising valorization route for oyster-shell waste and locally available volcanic resources. However, the environmental advantages of this approach remain to be quantitatively demonstrated through comprehensive life-cycle and techno-economic assessments. Therefore, the present work should be regarded as a proof of concept for waste valorization rather than evidence of environmental superiority over conventional materials. Consequently, the developed materials may have potential for high-value porous-material applications such as filtration, adsorption, and catalyst supports. However, both their functional performance and long-term durability remain to be experimentally verified before these applications can be realistically considered. Future work should therefore focus on reducing activator demand, assessing long-term durability, characterizing pore-network architecture and connectivity using dedicated porosity analysis techniques, and evaluating the functional performance of these materials in environmental applications. Additionally, future studies should investigate intermediate COS contents, particularly within the 2.5–7.5 wt% range, to more precisely determine the composition that maximizes pore development while maintaining adequate mechanical performance.

## Author contributions

Steve Keyrane Kuida Tchikapa: investigation, formal analysis, data curation, writing – original draft preparation, Priscilla S. Brunetto: supervision, funding acquisition, validation, writing – review & editing Antoine Scalabre: software, validation, writing – review & editing Placide Desiré Belibi Belibi: conceptualization, supervision Katharina M. Fromm: funding acquisition, supervision Julius Ndi Nsami: resources, supervision Antoine Elimbi: conceptualization, methodology, resources, supervision.

## Conflicts of interest

Authors have no competing interests to declare that are relevant to the content of this article.

## Abbreviations

VS: Volcanic slags
OS: Oyster shells
COS: Calcined oyster shells
L/S: Liquid/solid ratio
GVS_*x*_^*y*^COS: Geopolymers based on volcanic slag (VS) at molar concentration (*x* = 10 or 6 mol L^−1^) modified with *y* (0; 5; 10 or 15%) calcined oyster shells (COS)
WA: Water absorption

## Supplementary Material

RA-OLF-D6RA08514H-s001

## Data Availability

Authors declare that the data supporting the findings of this study are available within the paper and its supplementary information (SI) file. Supplementary information is available. See DOI: https://doi.org/10.1039/d6ra08514h.
