# Supplementary material for: Recycling oyster shell waste as a bio-derived pore-promoting additive for porous phosphate-based geopolymers produced from volcanic slag
Source: RSC Adv. 2026 Sep 28;16(45):57914–30. doi: 10.1039/d6ra08514h (PMC13618061; doi:10.1039/d6ra08514h)
Supplement: Supplementary file 1 [file RA-016-D6RA08514H-s001.pdf]

Supplementary Information (SI) for RSC Advances.
This journal is © The Royal Society of Chemistry 2026

Supporting Information

Recycling Oyster Shell Waste as a Bio-Derived Pore-Promoting Additive for Porous Phosphate-Based Geopolymers Produced from Volcanic Slag

Steve Keyrane Kuida Tchikapa[1,*], Priscilla Brunetto[2], Antoine Scalabre[2], Placide Desiré Belibi Belibi [1,*], Katharina M. Fromm[2], Julius Ndi Nsami[1] and Antoine Elimbi [3]

[1]*Applied Physical and Analytical Chemistry Laboratory, Department of Inorganic Chemistry, University of Yaoundé I, Yaoundé, Cameroon*
[2]*Department of Chemistry, University of Fribourg, Chemin du Musée, 9, 1700 Fribourg, Switzerland.*
[3]*Applied Inorganic Chemistry Laboratory, Department of Inorganic Chemistry, University of Yaoundé I, Yaoundé, Cameroon*
**Corresponding authors:* Placide Desiré Belibi Belibi [1,*] beplacide85@gmail.com
Steve Keyrane Kuida Tchikapa[1,*] keyranekuida70@gmail.com

Text S1. Raw materials

Volcanic slag

Volcanic slag (VS) was collected from the Adamawa region of Cameroon and used as the aluminosilicate precursor for geopolymer synthesis. Prior to use, the material was oven-dried at 105 ˚C for 24 h to remove residual moisture, crushed, and sieved to obtain a particle size fraction below 100 µm. The resulting powder was subsequently used for all geopolymer formulations.

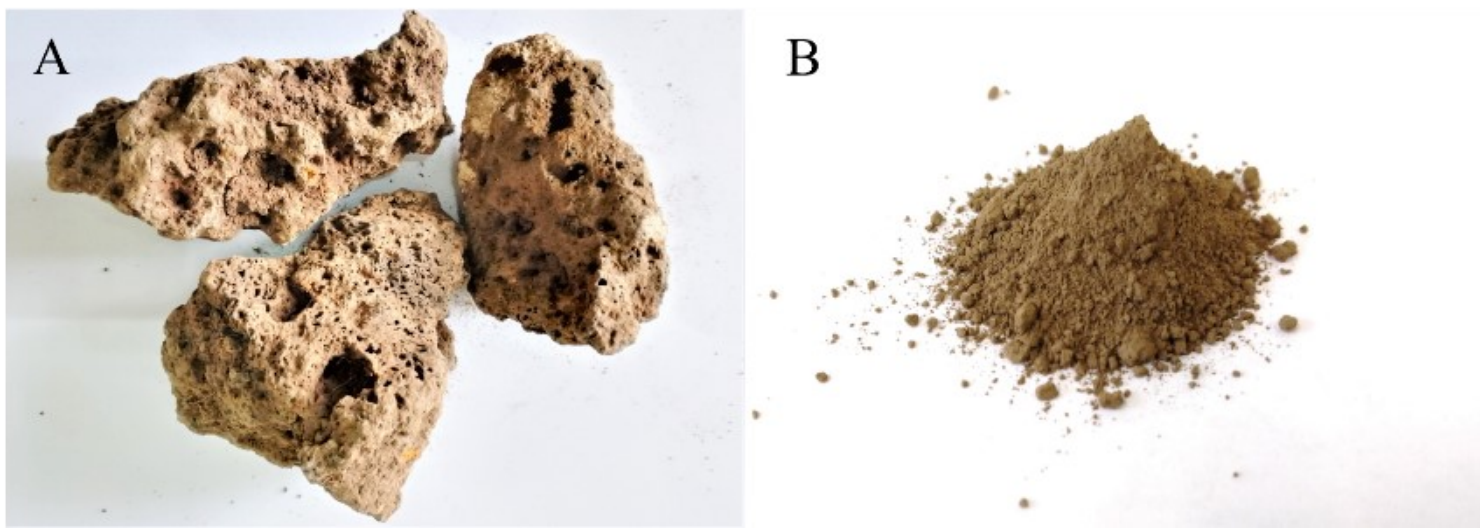

Figure S1: Volcanic slag used as geopolymer precursor: (A) raw volcanic slag and (B) ground volcanic slag powder (<100 µm).

Oyster shells

Oyster shells (OS) were collected from the coastal area of Mouanko (Cameroon). The shells were thoroughly washed with distilled water, dried at 105 °C for 24 h, and calcined at 900 ˚C for 4 h using a heating rate of 5 °C min^{-1} in a programmable electric furnace (*Nabertherm LH 60/14*). The calcined product was subsequently ground and sieved below 100 µm to obtain the calcined oyster shell powder (COS) used as a calcium-rich additive capable of promoting pore development in the geopolymer formulations.

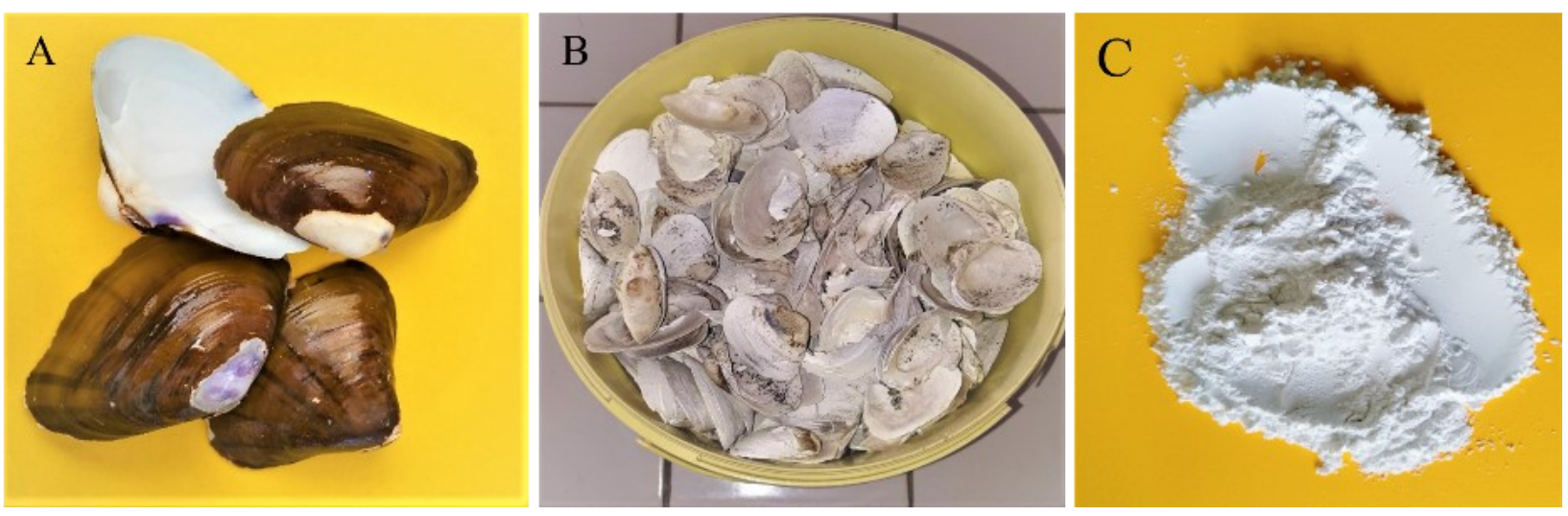

Figure S2: Oyster-shell processing route: (A) raw oyster shells, (B) calcined oyster shells, and (C) calcined oyster shell powder (COS).

Text S2. Characterization methods

X-Ray Fluorescence spectroscopy (XRF)

The chemical compositions of VS and COS were determined by wavelength-dispersive X-ray fluorescence spectroscopy (XRF, *Malvern PANalytical Zetium*). The oxide compositions are reported in Table S1.

Table S1: Chemical composition (wt.%) of volcanic slag (VS) and calcined oyster shells (COS).

OXYDES	SiO_2	Al_2O_3	Fe_2O_3	CaO	K_2O	MgO	Na_2O	MnO	TiO_2	P_2O_5
VS	40.89	19.45	16.69	7.68	0.577	7.85	0.97	0.26	3.84	0.96
COS	0.30	0.19	0.19	74.73	/	0.86	0.57	/	/	/

Specific surface area and pore size distribution (BET-BJH)

The specific surface area and pore characteristics of VS were determined from N_2 adsorption-desorption isotherms using a *Micromeritics 3Flex analyzer*. Surface area values were calculated according to the Brunauer-Emmett-Teller (BET) method, whereas pore characteristics were obtained using the Barrett-Joyner-Halenda (BJH) model. BET-BJH analysis was performed exclusively on the VS to characterize its initial textural properties. No quantitative porosity measurements were performed on the cured geopolymer samples. Consequently, the discussion of pore development in the present work is primarily based on SEM observations, water absorption measurements, and phase assemblage analysis.

The resulting parameters are summarized in Table S2. According to the IUPAC classification, micropores correspond to pore diameters below 2 nm, mesopores range from 2 to 50 nm, and macropores exceed 50 nm.

Table S2: Surface area and pore characteristics of VS determined by BET-BJH analysis.

Sample	BET surface area	Langmuir surface area	Micropore volume	Micropore area	Median pore width	Maximum pore volume
	($m^2.g^{-1}$)	($m^2.g^{-1}$)	($cm^3.g^{-1}$)	($m^2.g^{-1}$)	(nm)	($cm^3.g^{-1}$)
VS	44 ± 1	53.05 ± 0.26	0.017	40.21	0.55	0.023

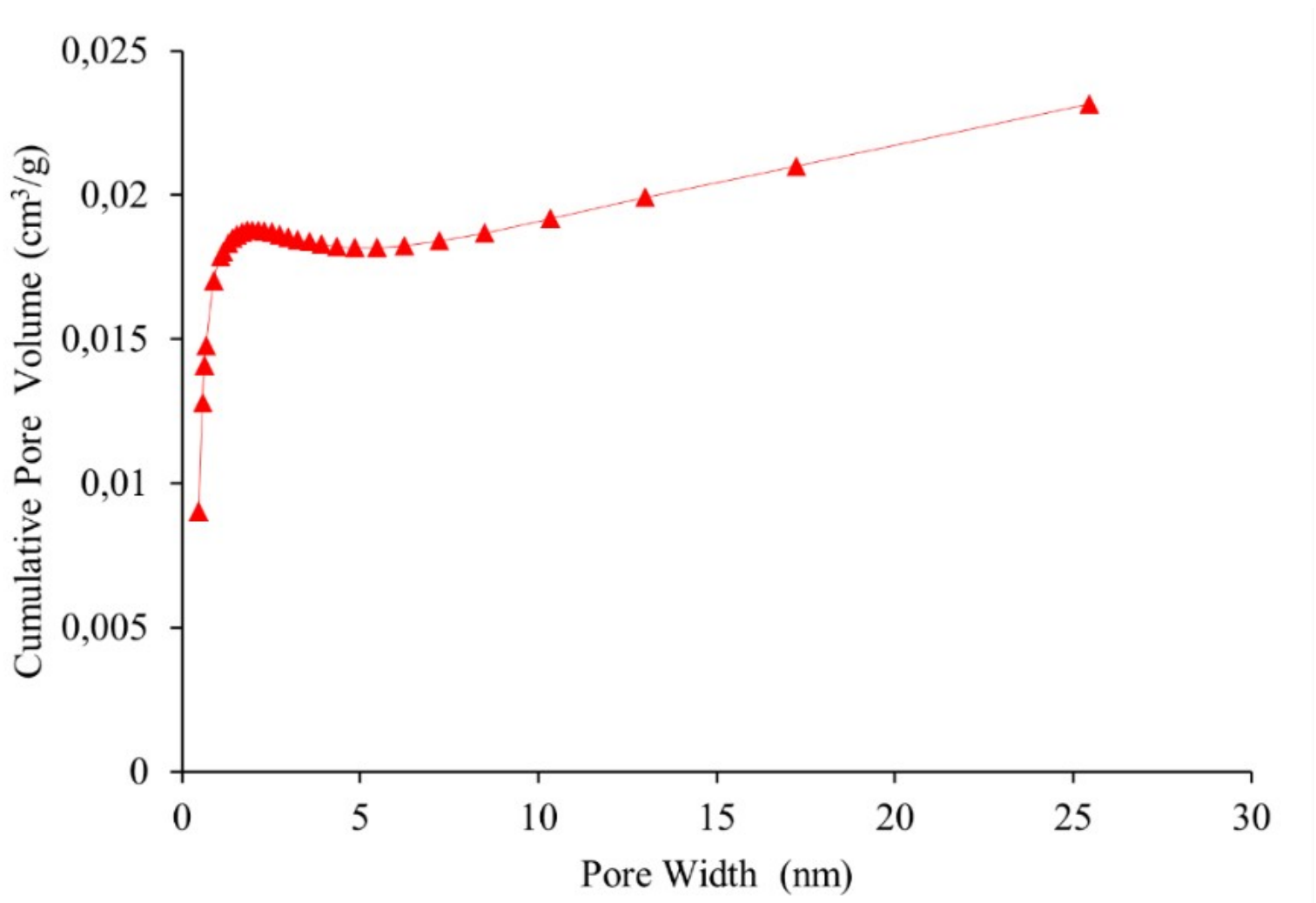

Figure S3: Pore-size distribution of volcanic slag determined from nitrogen adsorption-desorption measurements.

Thermal analysis (TGA/SDTA)

Thermogravimetric and differential thermal analyses (TGA/SDTA) were carried out using a *Mettler Toledo TGA/DSC 3+* instrument. Measurements were performed to evaluate the thermal stability of volcanic slag and determine the optimum calcination conditions for converting calcium carbonate contained in oyster shells into reactive calcium-rich phases suitable for geopolymer synthesis.

Fourier Transform Infrared (FT-IR)

Fourier-transform infrared spectra were recorded using a PerkinElmer Frontier spectrometer operating in transmittance mode over the spectral range 4000–400 cm^{-1}. FTIR analysis was used to identify the principal chemical bonds present in the raw materials and reaction products formed during geopolymerization.

X-Ray Diffraction (XRD)

Phase identification was performed using X-ray diffraction (*Bruker D8 Advance*, CuK_α radiation, λ = 1.5406 Å). Diffraction patterns were analyzed using *Match!* software (Crystal Impact, Version 4), and crystalline phases were identified by comparison with entries from the Crystallography Open Database (COD). Monetite identification in the geopolymer samples was established from the presence of multiple characteristic reflections observed consistently across the different formulations.

Text S3. Mechanical tests

Setting time

Setting-time measurements were performed on fresh paste obtained from the same formulation batch immediately after mixing using a Vicat apparatus according to the EN 196-3 standard at 23 ± 5°C [S4].

Water absorption

Water absorption was calculated using Eq. (1) according to ASTM C373-88 [S1]:

$$WA\% = \frac{(W_w - W_d)}{W_d} \times 100 \quad (1)$$

Where WA% is the absorption of water (%), W_w and W_d are the mass (g) of water-saturated and dry specimens, respectively. Fresh geopolymer pastes were cast into dedicated cylindrical molds (20 mm diameter × 40 mm height) specifically prepared for water-absorption measurements. Three independent cylindrical specimens were tested for each formulation. After 28 days of curing, cylindrical specimens were dried at 105 °C for 24 h and weighed before and after immersion in water for 24 h.

Linear shrinkage

Linear shrinkage was determined after 28 days of curing according to ASTM C157 using Eq. (2) [S2, S5] :

$$Lc = \frac{L_0 - L_{28}}{L_0} \times 100 \quad (2)$$

Where L_c = Linear shrinkage (%), L_{28} = length after 28 days of curing (mm), L_0 = length at the demolding day (mm).

Compressive strength

Compressive strength tests were performed on 28-day-old cubic specimens (40 × 40 × 40 mm) using a 250 kN electrohydraulic testing machine (*Impact Test Equipment Ltd.*) at a loading rate of 0.5 MPa s^{-1}. Compressive strength was calculated using Eq. (3) [S3] :

$$\sigma = \frac{F}{A} \tag{3}$$

Where F = applied load (N) and A = loaded surface area (mm^2) and σ = compressive strength (MPa).

All measurements were performed in triplicate, using three independent specimens per formulation. Reported values correspond to mean values, and the error bars shown in the figures represent the corresponding standard deviations.

Scanning Electron Microscopy/ Energy Dispersive Spectroscopy (SEM/EDS)

The microstructure of the synthesized geopolymers was investigated by scanning electron microscopy coupled with energy-dispersive spectroscopy (SEM/EDS). Specimens were sectioned using a precision low-speed saw (*Buehler Isomet*®), carbon-coated using a *BALZERS CED 030* carbon evaporator, and rendered conductive with a silver-based conductive coating. Observations were carried out using a *FEI Thermo Fisher XL30 FEG SEM* equipped with an *X-MAX 150 mm^2 silicon drift detector (SDD)* with an energy resolution of 127 eV.

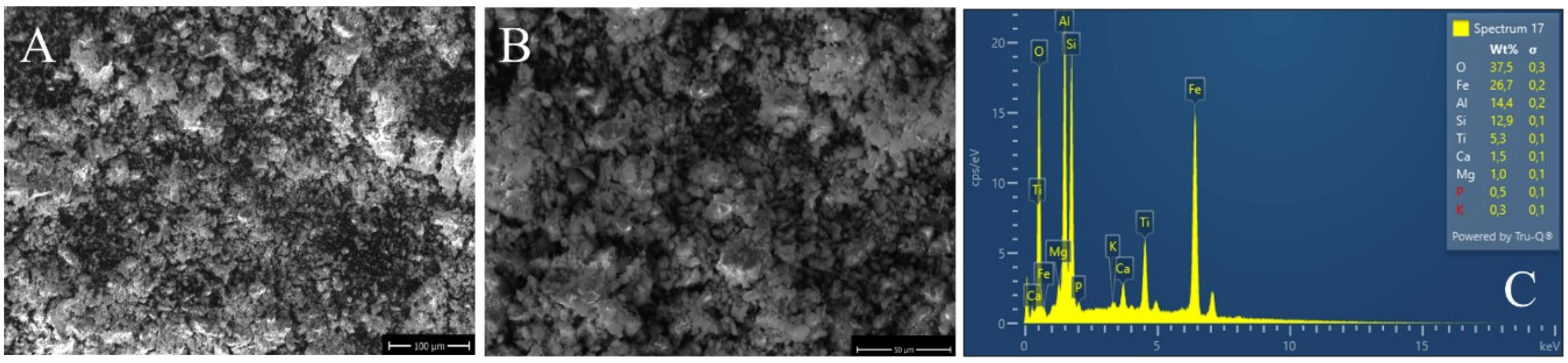

Figure S4: SEM and EDS analyses of volcanic slag (VS).

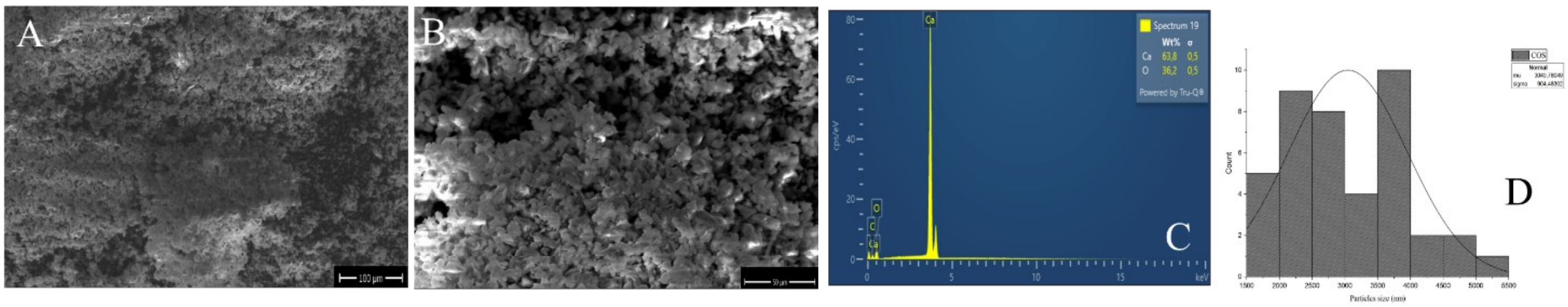

Figure S5: SEM and EDS analyses of calcined oyster-shell powder (COS), together with particle-size estimation derived from image analysis.

Particle-size measurements were performed using ImageJ-Fiji software. The average particle size determined from SEM images was approximately 3.04 µm.

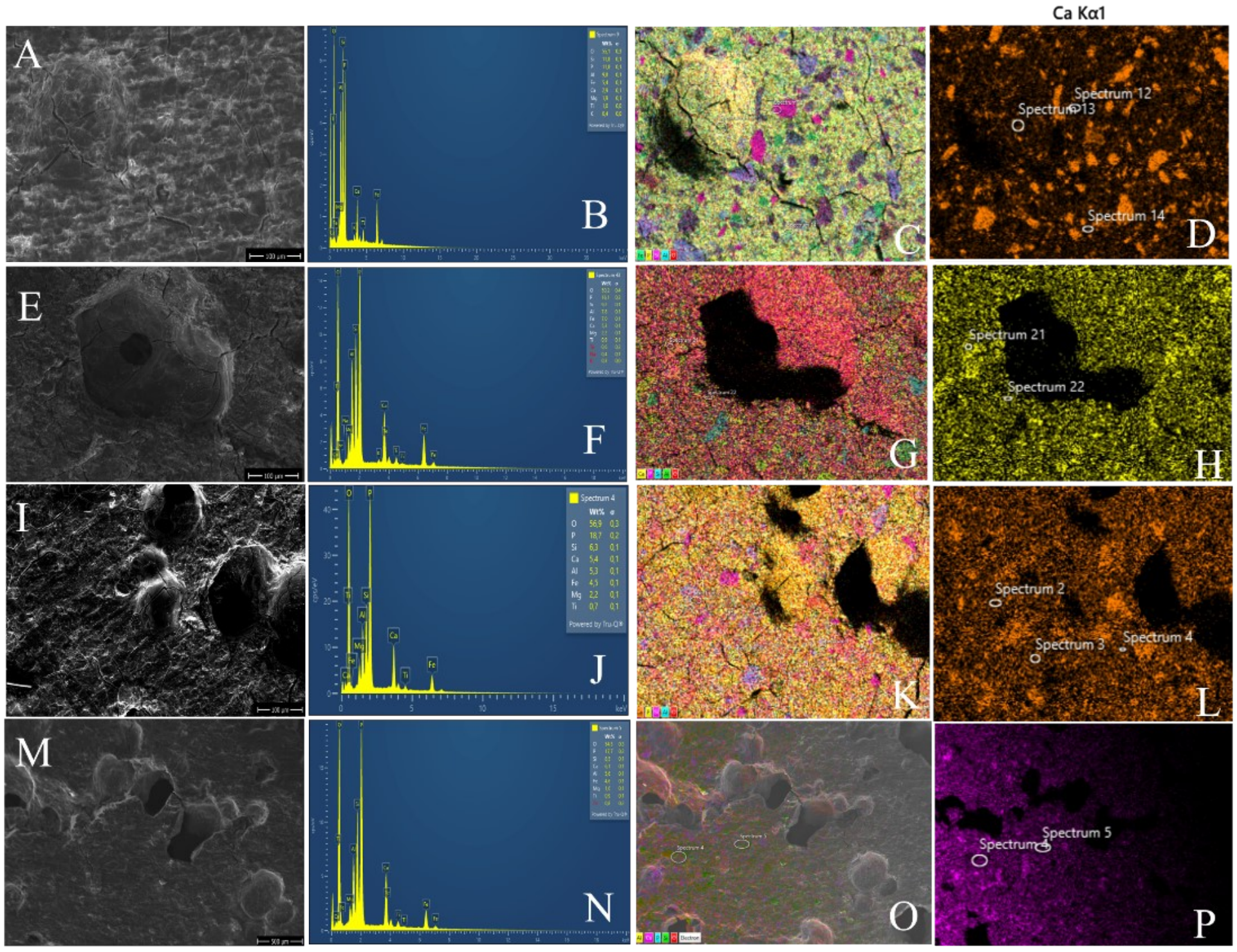

Figure S6: SEM images and EDS elemental mapping of calcium distribution in $GVS_{10}{}^{0}COS$ (A. B. C. D). $GVS_{10}{}^{5}COS$ (E. F. G. H). $GVS_{10}{}^{10}COS$ (I. J. K. L). $GVS_{10}{}^{15}COS$ (M. N. O. P). The elemental maps are presented for qualitative assessment of calcium distribution and do not provide quantitative chemical information.

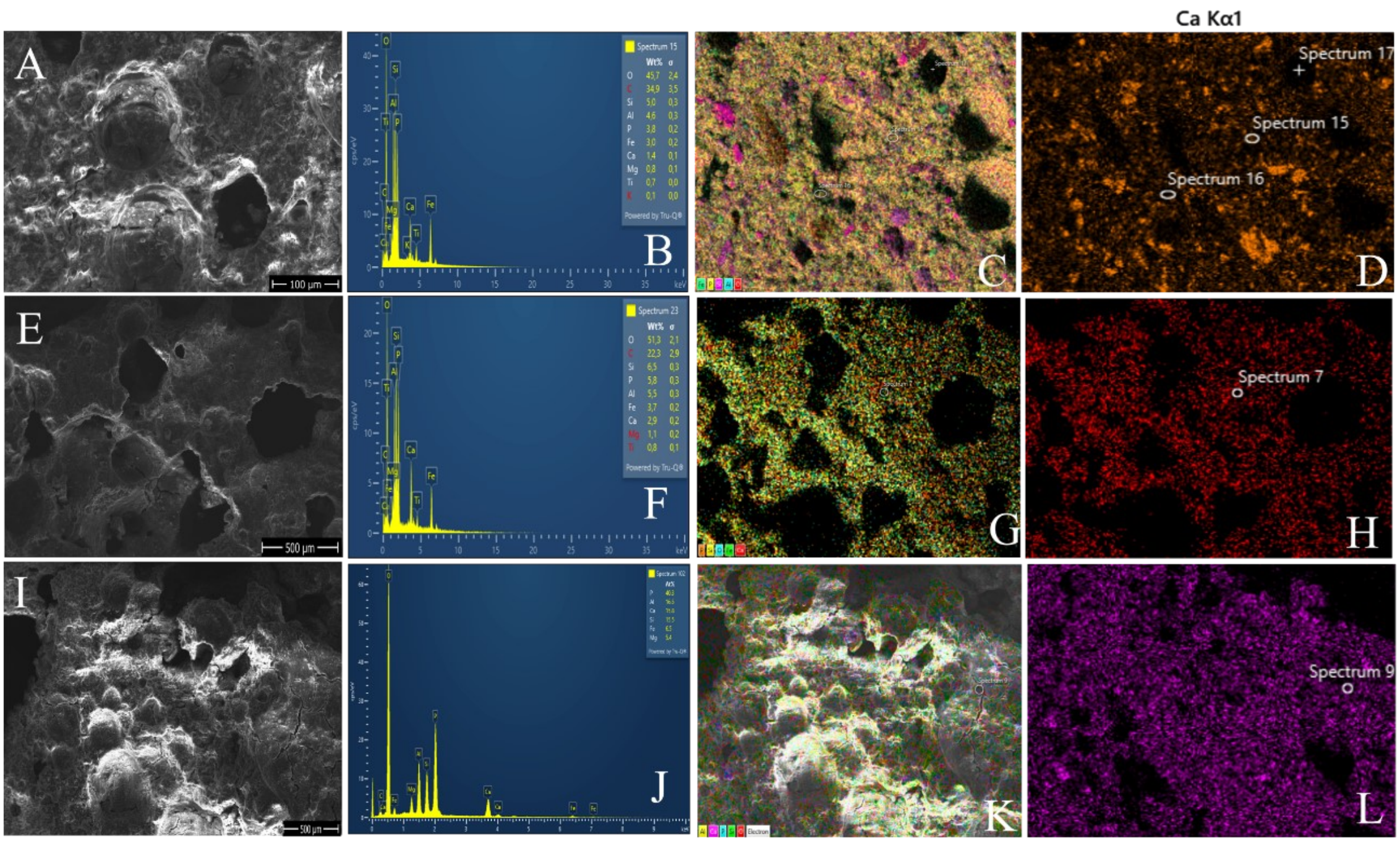

Fig S7. SEM images and EDS elemental mapping of calcium distribution in $GVS_{6}{}^{0}COS$ (A. B. C. D). $GVS_{6}{}^{10}COS$ (E. F. G. H). $GVS_{6}{}^{15}COS$ (I. J. K). The elemental maps are presented for qualitative assessment of calcium distribution and do not provide quantitative chemical information.

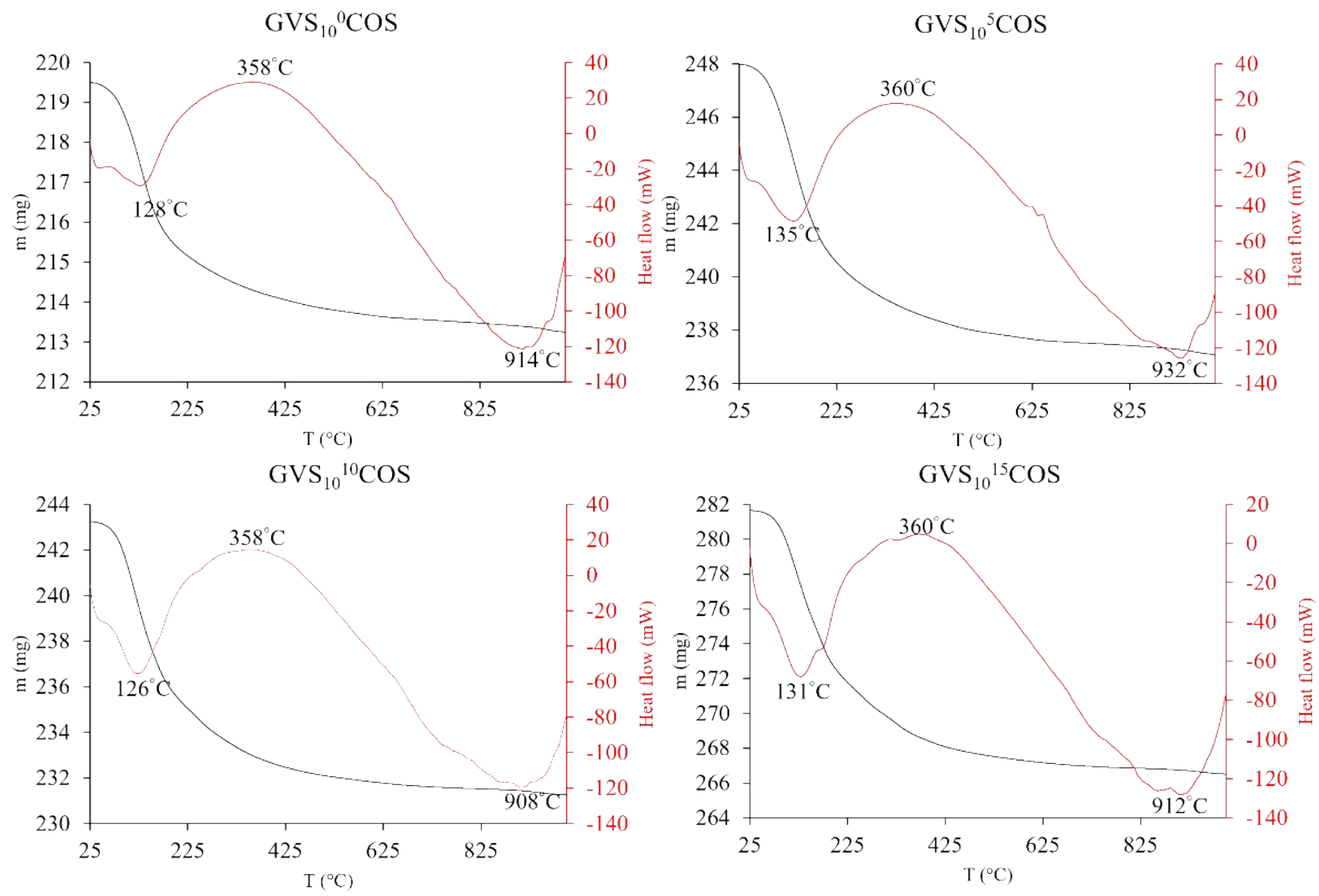

Fig S8. Thermal analyses of geopolymers synthesized using 10 mol L^{-1} phosphoric acid. ($GVS_{10}{}^{y}COS$).

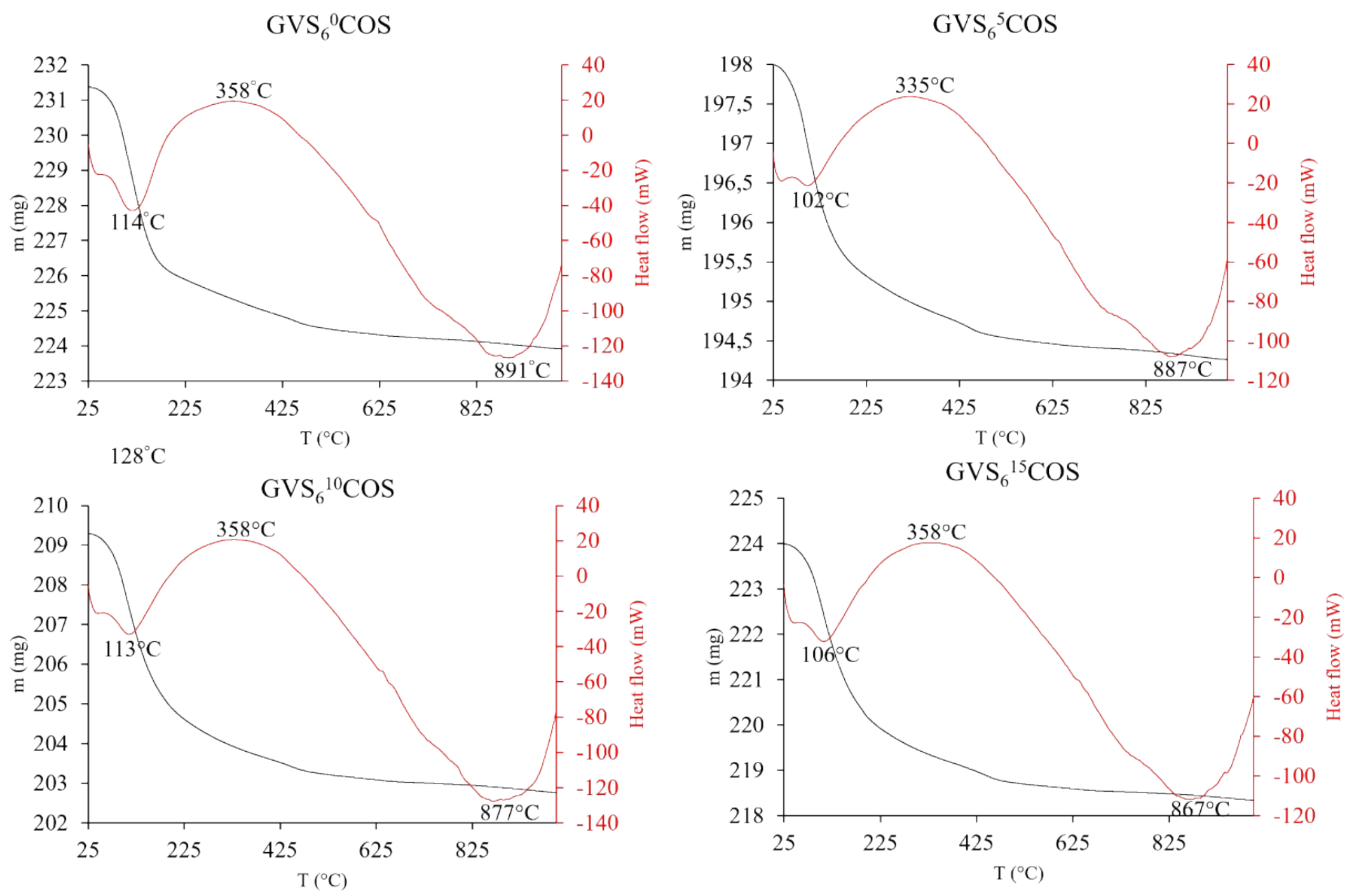

Fig S9. Thermal analyses of geopolymers synthesized using 6 mol L^{-1} phosphoric acid. ($GVS_{6}{}^{y}COS$).

Thermal analyses revealed that all geopolymer formulations remained thermally stable over the investigated temperature range. The thermograms show an endothermic event between approximately 100 and 120 °C associated with the release of physically adsorbed water,

followed by two exothermic events between approximately 330–360 ˚C and 860–900 ˚C, attributed to structural rearrangements within the geopolymeric network.

Because the incorporation of COS increased the water demand of the mixtures, different L/S ratios were required to obtain workable pastes. Consequently, COS content and L/S ratio were varied simultaneously, and the reported physicomechanical properties should be interpreted as reflecting their combined influence.

Table S3: Values of different parameters studied. Values correspond to the mean of three independent specimens tested for each formulation.

Concentration of the activating phosphoric acid solution	*Formulations*	*Setting Time (min)*	*Water Absorption (%) (WA%)*	*Linear Shrinkage (%) (Lc)*	*Compressive Strength (MPa)*
10	$GVS_{10}^{0}COS$	409 ± 2.83	11.03 ± 0.47	1.55 ± 0.34	22.85 ± 1.38
	$GVS_{10}^{5}COS$	49 ± 5.65	10.15 ± 0.12	0.80 ± 0.18	21.74 ± 1.25
	$GVS_{10}^{10}COS$	23 ± 0	8.53 ± 1.06	0.25 ± 0.01	7.42 ± 0.55
	$GVS_{10}^{15}COS$	15 ± 5.65	6.29 ± 0.14	0.19 ± 0.22	5.47 ± 0
6	$GVS_{6}^{0}COS$	85 ± 7.07	19.76 ± 0.31	0.11 ± 0.014	9.24 ± 0.45
	$GVS_{6}^{5}COS$	6 ± 0	33.37 ± 3.26	0.27 ± 0.006	5.47 ± 0.55
	$GVS_{6}^{10}COS$	5.5 ± 3.5	38.81 ± 0.68	0.34 ± 0.02	1.17 ± 0
	$GVS_{6}^{15}COS$	4 ± 0	59.23 ± 0.84	0.85 ± 0.12	0.62 ± 0.02

Supporting Information References

[S1] R. C. Kaze. L. M. Beleuk à Moungam. M. Cannio. R. Rosa. . Kamseu. U. C. Melo. C. Leonelli. “Microstructure and engineering properties of $Fe_2O_3(FeO)$-Al_2O_3-SiO_2 based geopolymer composites.” *J. Clean. Prod.*. vol. 3. no. July. 2018. doi: 10.1016/j.jclepro.2018.07.171.

[S2] O. O. Judicaël Geferson. B. B. Placide Desire. E. J. Serge. A. N. Odogu. N. J. Nsami. and E. Antoine. “Sustainable and cleaner production of a porous geopolymer support based on metakaolin and natural calcium oxide.” *Journal of Building Engineering*. vol. 96. Nov. 2024. doi: 10.1016/j.jobe.2024.110638.

[S3] M. A. H. Bhuyan and T. Luukkonen. “Adsorption of methylene blue by composite foams containing alkali-activated blast furnace slag and lignin.” *International Journal of Environmental Science and Technology*. vol. 21. no. 4. pp. 3789–3802. Feb. 2024. doi: 10.1007/s13762-023-05245-5.

[S4] EN. T. S. 196-3; Methods of Testing Cement—Part 3: Determination of Setting Times and Soundness. *Turkish Standards Institution : Ankara. Turkey*. 2017.

[S5] ASTM. Standard Test Method for Length Change of Hardened Hydraulic-Cement Mortar and Concrete; ASTM International: West Conshohocken. PA. USA. 2017.
